# Multicenter analysis of sputum microbiota in tuberculosis patients

**DOI:** 10.1371/journal.pone.0240250

**Published:** 2020-10-12

**Authors:** Claudia Sala, Andrej Benjak, Delia Goletti, Sayera Banu, Jesica Mazza-Stadler, Katia Jaton, Philippe Busso, Sille Remm, Marion Leleu, Jacques Rougemont, Fabrizio Palmieri, Gilda Cuzzi, Ornella Butera, Valentina Vanini, Senjuti Kabir, S. M. Mazidur Rahman, Laurent Nicod, Stewart T. Cole

**Affiliations:** 1 Faculty of Life Sciences, Ecole Polytechnique Fédérale de Lausanne, Lausanne, Switzerland; 2 National Institute for Infectious Diseases “L. Spallanzani”-IRCCS, Rome, Italy; 3 icddr,b, Dhaka, Bangladesh; 4 Centre Hospitalier Universitaire Vaudois, Lausanne, Switzerland; 5 Swiss Institute of Bioinformatics (SIB), Lausanne, Switzerland; 6 BioInformatics Competence Center, UNIL-EPFL, Lausanne, Switzerland; 7 Department of Theoretical Physics, University of Geneva, Geneva, Switzerland; University of Cape Town, SOUTH AFRICA

## Abstract

The impact of tuberculosis and of anti-tuberculosis therapy on composition and modification of human lung microbiota has been the object of several investigations. However, no clear outcome has been presented so far and the relationship between *M*. *tuberculosis* pulmonary infection and the resident lung microbiota remains vague. In this work we describe the results obtained from a multicenter study of the microbiota of sputum samples from patients with tuberculosis or unrelated lung diseases and healthy donors recruited in Switzerland, Italy and Bangladesh, with the ultimate goal of discovering a microbiota-based biomarker associated with tuberculosis. Bacterial 16S rDNA amplification, high-throughput sequencing and extensive bioinformatic analyses revealed patient-specific flora and high variability in taxon abundance. No common signature could be identified among the individuals enrolled except for minor differences which were not consistent among the different geographical settings. Moreover, anti-tuberculosis therapy did not cause any important variation in microbiota diversity, thus precluding its exploitation as a biomarker for the follow up of tuberculosis patients undergoing treatment.

## Introduction

Tuberculosis (TB) is a widespread infectious disease caused by *Mycobacterium tuberculosis* which is transmitted through aerosol droplets containing bacilli released from infected individuals. In 2018, the World Health Organization estimated 10 million new TB cases worldwide that led to 1.6 million fatalities, thus ranking TB as the main cause of death from a single pathogen [1].

*M*. *tuberculosis* pathogenesis and outcome of TB infection are impacted by several factors such as bacterial lineage [2], drug susceptibility of the strains [3], co-infection with HIV [4] or Cytomegalovirus (CMV) [5], comorbidities like diabetes [6], genetic predisposing factors (for instance IFN-γ/IL-12 deficiency [7]) and malnutrition [8].

One emerging host factor that may play a role in TB disease is the microbiota, which is defined as the microbial community that inhabits several sites of the body, including gut [9], vagina, lung and bladder [10–12]. Commensal microbial communities live in these mucosal compartments and are believed to positively influence human health, by modulating the activity of the immune system, by enabling the elimination of ineffectively working immune cells and by protecting the host from pathogens [11,13]. Disruption of the microbiota (dysbiosis) caused by antibiotic treatment, nutritional factors or infections may lead to dysfunction of the respective organs [14], thereby promoting disease. It has been shown that antibiotic therapy can modify the composition of the microbiota and, in some cases, is associated with adverse health effects in patients [15].

Anti-TB treatment is long (at least six months for drug-susceptible cases) and involves a combination of narrow- as well as broad-spectrum drugs in regimens that may impact the structure and composition of the microbial communities co-existing within the host. First-line anti-TB treatment (composed of isoniazid, rifampicin, ethambutol and pyrazinamide) was shown to have little effect on microbiota composition in the gut of TB patients, although the relative abundance of some taxa (*Lactobacillus* and *Prevotella*) was found to be altered [16].

Studies conducted in the murine model of TB revealed that colonization of the gut by *Helicobacter hepaticus* results in increased lung inflammation and pathology upon challenge with *M*. *tuberculosis* [17,18]. On the other hand, it seems that *H*. *pylori* has the opposite effect [19]. Unfortunately, few investigations have been carried out so far in humans and these have had contradictory results. One study reported no significant alteration in gut microbiota in individuals with latent TB infection [16] while another found changes in the abundance of short-chain fatty acid-producing bacteria [20,21]. Interestingly, a study focused on one multidrug-resistant TB case sampled during therapy discovered that the gut flora was depleted by long-term treatment with second-line drugs [22]. Altogether, these data suggest the existence of an interplay between commensal gut microbes and lung morbidities via the gut-lung axis. Specifically, metabolites produced by intestinal bacteria impact immune responses in the respiratory tract as in the case of allergic asthma [23–25].

The interplay between microbiota of the respiratory tract and TB has been the object of various analyses, reviewed in [26–28]. Most of the human studies have been performed on sputum samples or throat swabs and oropharyngeal secretions by means of 16S ribosomal DNA (rDNA) amplification and sequencing. Overall, the findings are limited and inconsistent, with high variability measured in different experimental settings. For example, Cui and colleagues showed that active TB disease is associated with higher taxon diversity [29]. Another group found few differences between TB cases and controls, limited to *Mogibacterium*, *Moryella* and *Oribacterium* [30]. A third investigation reported that *Streptococcus*, *Prevotella* and *Neisseria* spp. were more abundant in TB patients than in the healthy cohort [31]. On the contrary, Wu and co-workers discovered that *Prevotella* was reduced in TB cases compared to healthy controls [32], whereas Botero compared bacterial and fungal taxa found in sputum specimens to those in nasal swabs in TB patients and reported that these were distinct [33]. On the other hand, a study based on bronchoalveolar lavage discovered more *Cupriavidus*, *Mycobacterium* and *Porphyromonas* in people with TB lesions and higher abundance of *Streptococcus* in controls [34].

In this work we analyzed the taxonomic composition of sputum microbiota in TB patients and in control subjects (either healthy donors or patients with lung disease different from TB) by means of 16S rDNA amplification from sputum samples. We report patient-specific microbial communities, which precluded extrapolating common signatures. Additionally, antibiotic TB treatment did not appear to drive any alterations in sputum microbiota composition or relative species abundance, thereby preventing the definition of a microbiota-based biomarker.

## Results

### Rationale and aim of the work

Previously, multiple studies have demonstrated how microbiota composition in gut, lungs and skin impacts the clinical evolution of various pathologies, including inflammatory, auto-immune and behavioral disorders as well as infectious diseases [35–40]. In this multicenter study, we investigated the composition of sputum microbiota in TB disease and during anti-TB treatment. More specifically, we recruited patients affected by pulmonary TB in different countries (mainly Bangladesh, Italy and Switzerland), thereby assessing the sputum microbiota of different ethnic cohorts, and performed bacterial 16S rDNA amplification from their sputum samples, followed by high-throughput sequencing. Our objectives comprised the following:

- Analyze and compare the composition of sputum microbiota in TB patients and in control subjects, not affected by TB disease but either healthy or affected by pneumonia.
- Characterize the sputum microbiota in TB patients undergoing anti-TB therapy. In this case, a time-course study was undertaken.

The overall goal of the study was the definition of a new biomarker, based on microbiota composition.

Since patients were recruited in different countries at different times and may have been subjected to different therapeutic protocols, we anticipated that a unique bioinformatic analysis might be impossible to carry out and, most importantly, this would be scientifically weak. While DNA preparation, 16S rDNA PCR amplification, library preparation and sequencing followed the same protocols throughout the study, bioinformatic tools were optimized while the study was ongoing. For these reasons, results will be presented separately.

### First analyses of microbiota composition in sputum samples received from FIND

To explore the diversity of sputum microbiota in TB patients and to compare it to that of patients not affected by TB disease, we collaborated with FIND (Foundation for Innovative New Diagnostics, Geneva, Switzerland), which provided access to the first samples analyzed. The first batch of sputum samples included those obtained from 15 patients with active TB and from 15 individuals without TB. None of these individuals were diagnosed as HIV positive. Non-TB samples were from patients coming from Canada (Winnipeg), The Gambia, Spain (Barcelona), whereas Vietnam, Brazil (Salvador), The Gambia and Uganda represented the countries where TB patients were sampled (Table 1).

**Table 1:** Characteristics of the first batch of samples received from FIND.

		Pulmonary TB	Non-TB samples	Total
Sputum samples (%)		15 (50)	15 (50)	30 (100)
**Median age in years (range)**		37 (22–74)	48 (18–78)	43 (18–78)
**Female gender (%)**		5 (33)	5 (33)	10 (33)
**Origin (%)**	Vietnam	8 (53)	0	8 (27)
	Brazil(Salvador)	2 (13)	0	2 (7)
	Uganda	4 (27)	0	4 (13)
	The Gambia	1 (7)	5 (33)	6 (20)
	Canada(Winnipeg)	0	5 (33)	5 (17)
	Spain (Barcelona)	0	5 (33)	5 (17)
**BCG N (%)**	Vaccinated	8 (53)	3 (20)	11 (37)
	Unvaccinated	1 (7)	7 (47)	8 (27)
	Unknown status	6 (40)	5 (33)	11 (37)
**Smokers (%)**		4 (27)	5 (33)	9 (60)
**Chronic alcoholism (%)**		5 (33)	1 (7)	6 (20)

TB: Tuberculosis; BCG: Bacillus Calmette–Guérin vaccine. Information about BCG vaccination, smoking and alcoholism was collected from clinical records.

**Fig 1:**
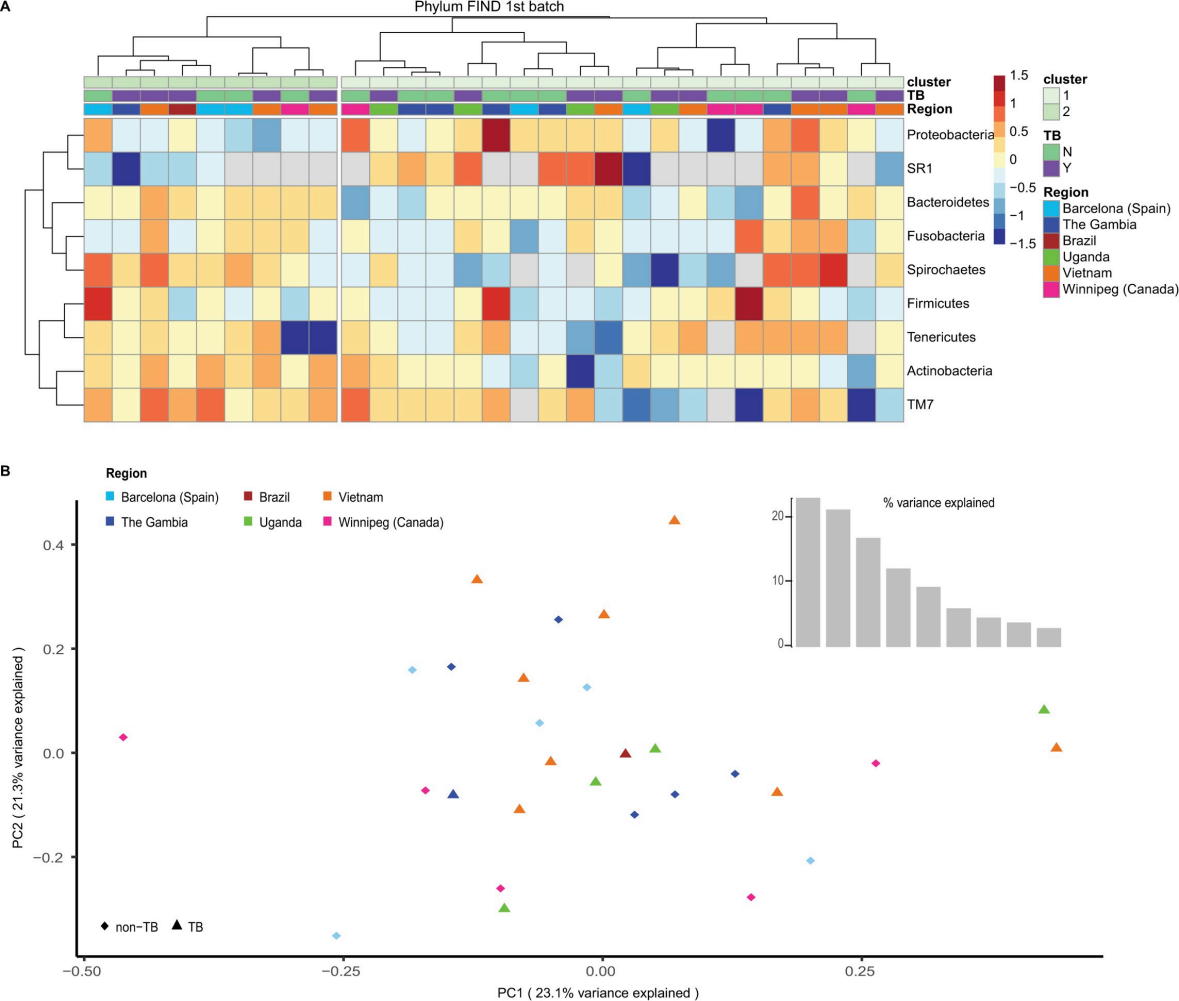
Analysis of the first batch of samples received from FIND. **A.** Heatmap of Phylum abundances across samples. Hierarchical clustering did not segregate the data according to disease (TB in violet, non-TB in green) nor to the region of origin. **B.** PCoA (PC1 vs. PC2) displaying all of the samples, colored according to the country. The different shapes (triangle or circle) indicate TB and non-TB samples, respectively. No segregation could be observed. The percentage of variance explained for the first components is shown in the inset.

The first parameter considered in the analysis was alpha diversity, which represents a measure of the taxonomic diversity of the samples and counts the number of distinguishable taxa in each sample [41]. No significant difference was observed, indicating that TB and non-TB specimens were equally diverse and there was no sample with a greater number of taxa identified (S1A Fig). When normalized Phylum abundances in the various samples were considered, no hierarchical clustering was noted according to the disease (TB or non-TB) or to the region of origin, therefore indicating high variability (Fig 1A). Similarly, Principal Component Analysis (PCoA) did not display any segregation of the sputum samples (Fig 1B).

**Fig 2:**
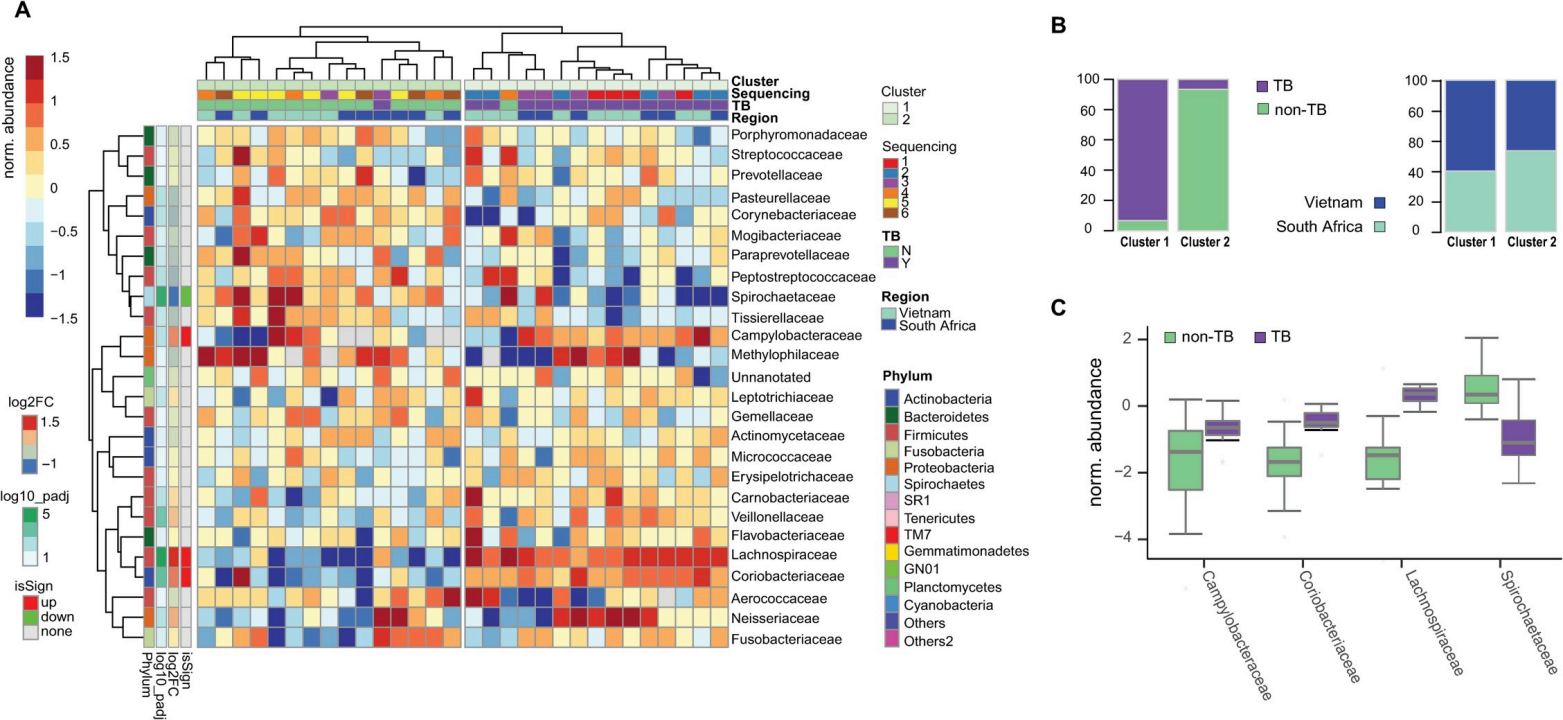
Analysis of the second batch of samples received from FIND. A. Heatmap of Family abundances across samples. The hierarchical clustering shows a segregation for the majority of the TB (violet) and non-TB samples (green). No segregation is observed for the sequencing batch or the region of origin. Annotations of rows provide the strengths and significances of Families in TB samples compared to non-TB samples (differential abundance analysis–see methods - significant if absolute log2 Fold-Change > 1 and adjusted p-value < 0.05). **B.** Barplots quantifying the representations of TB and non-TB samples in the two clusters obtained in **A** as well as their region of origin. **C.** Abundance distributions of representative Families identified in **A** in TB and non-TB samples.

The study was further extended to a geographically matched dataset that included 30 HIV negative patients, 15 of which with active TB and 15 without. In each of the two groups, 8 samples came from Vietnam and 7 from South Africa (Table 2). As found in the previous unmatched analysis, alpha diversity was similar for the two groups at all taxonomic levels (S1B Fig). Analysis of taxonomic composition by hierarchical clustering revealed clear segregation of TB and non-TB samples at the Family level whereas no impact of the country of origin was noticed (Fig 2A and 2B and S2A Fig). Interestingly, the sequencing batch had a minor effect, if any, on the results obtained (Fig 2A and S2A Fig). PCoA partially segregated bacterial Families belonging to Bacteroidetes from those belonging to Fusobacteria on PC1 and Proteobacteria Families from Firmicutes Families on PC2 (S2B Fig), suggesting differences in the abundance profiles for these taxa. Furthermore, differential abundance analysis revealed Lachnospiraceae (Phylum Firmicutes) as one of the Families characterized by absolute log2 fold-change greater than 1 and adjusted p-value lower than 0.05 (Fig 2C).

**Table 2:** Characteristics of the second batch of samples received from FIND.

		Pulmonary TB	Non-TB samples	Total
Sputum samples (%)		15 (50)	15 (50)	30 (100)
**Median age in years (range)**		40 (22–61)	44 (29–72)	41 (22–72)
**Female gender (%)**		3 (20)	3 (20)	6 (20)
**Origin (%)**	Vietnam	8 (53)	8 (53)	16 (53)
	South Africa	7 (47)	7 (47)	14 (47)
**BCG N (%)**	Vaccinated	7 (47)	8 (53)	15 (50)
	Unvaccinated	5 (33)	4 (27)	9 (30)
	Unknown status	3 (20)	3 (20)	6 (20)
**Smokers (%)**		8 (53)	8 (53)	16 (53)
**Chronic alcoholism (%)**		4 (27)	4 (27)	8 (27)

TB: Tuberculosis; BCG: Bacillus Calmette–Guérin vaccine. Information about BCG vaccination, smoking and alcoholism was collected from clinical records.

### Microbial diversity in patients recruited in Switzerland

**Fig 3:**
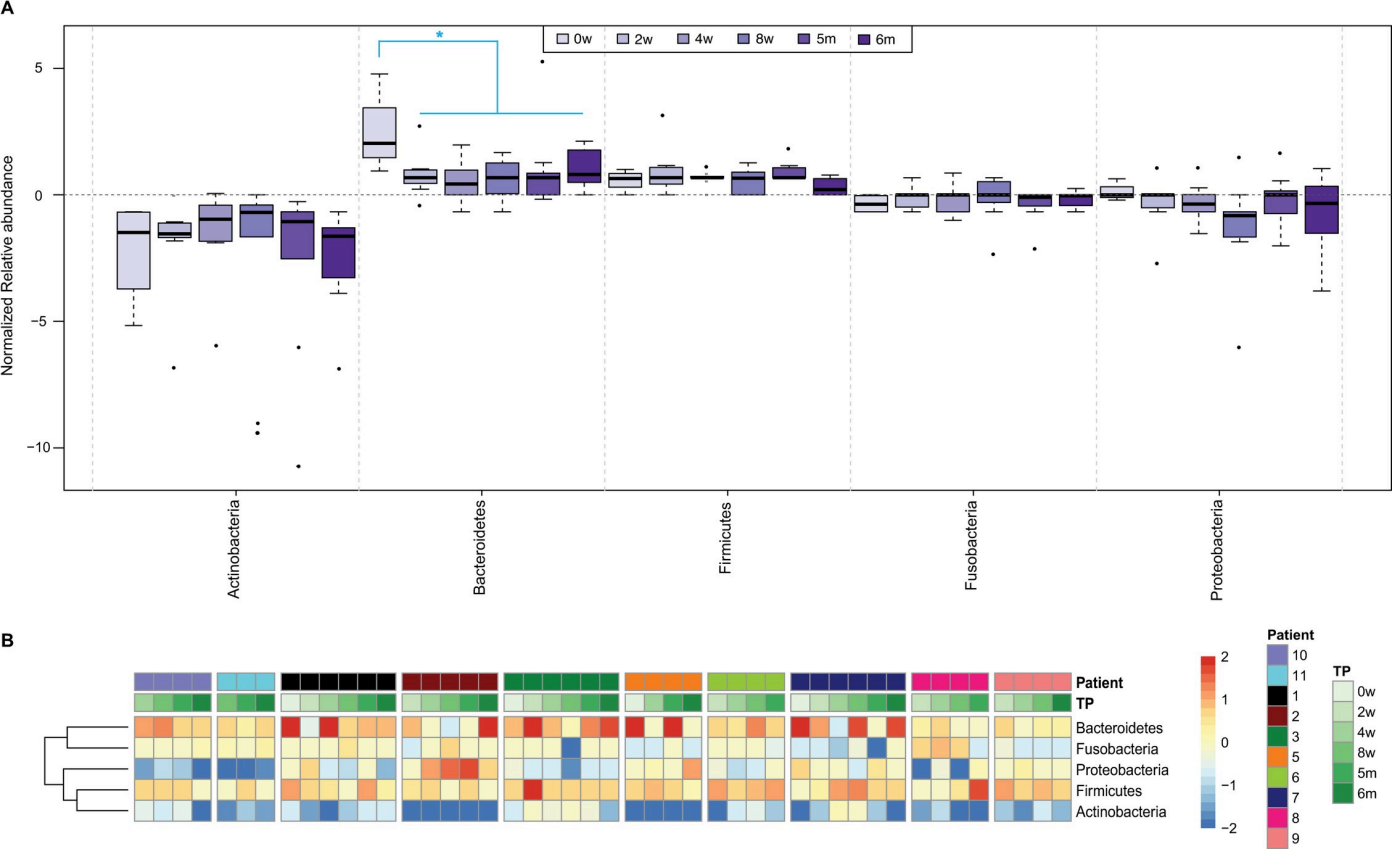
Analysis of samples received from CHUV. **A.** Phylum abundances per time-points. A significant change could only be observed for Bacteroidetes between time-point 0 and the other five time-points (pairwise t-test, with adjusted p-value < 0.01). **B.** Heatmap of Phylum abundances across samples and scaled by bacteria. Hierarchical clustering was applied to Phyla. Samples are ordered by patient (different colors) and by time-point (TP).

To extend the study to a different geographical setting and to characterize the evolution of sputum microbiota during anti-TB therapy, 10 TB patients from Switzerland were evaluated (Table 3). Induced sputum samples were collected at different time-points throughout therapy, i.e. 0, 2, 4, 8 weeks, 5 and 6 months. Actinobacteria, Bacteroidetes, Firmicutes, Fusobacteria and Proteobacteria represented the major Phyla identified in these samples (Fig 3A and 3B). When normalized Phylum abundances were calculated, a statistically significant change was observed for Bacteroidetes between time-point 0, which showed the highest level, and the other time-points (Fig 3A). On the other hand, the heatmap in Fig 3B illustrates the variability observed in Phylum abundances among the different patients and time-points. Detailed analysis of the mean and median values of the aggregated samples confirmed that Bacteroidetes, and the corresponding downstream taxonomic levels (i.e. Class Bacteroidia, Order Bacteroidales, Family Prevotellaceae, Genus Prevotella and Species *Prevotella melaninogenica*), were the predominant taxa at time-point 0 and their abundance gradually decreased with time (S3 Fig).

**Table 3:** Demographic characteristics of the subjects enrolled at the Centre Hospitalier Universitaire Vaudois (CHUV) in Lausanne, Switzerland.

		Pulmonary TB patients*
Enrolled subjects (%)		10 (100)
**Median age in years (range)**		38 (23–68)
**Female gender (%)**		6 (60)
**Origin (%)**	Western Europe (Switzerland, %)	3 (30)
	Eastern Europe (Romania, %)	2 (20)
	Africa (%)	4 (40)
	West Asia (%)	1 (10)
**Smokers (%)**		7 (70)

TB:Tuberculosis

*One patient was diagnosed with multi-drug resistant TB. Information about smoking was collected from clinical records.

### Microbial diversity in patients recruited in Italy

Recruitment of an additional set of samples at the National Institute for Infectious Diseases in Rome (Italy) allowed comparisons between patients affected by TB or by pneumonia (details are shown in Table 4). Overall, twenty-two patients were part of the study: 10 of these were diagnosed with active TB, whereas among the 12 patients without TB, 11 had pneumonia and 1 had a lung abscess. Sputa were collected from all of them at different time-points during therapy. Specifically, most of the TB patients were sampled at 0, 2 weeks, 4 weeks, 8 weeks, 5 months and 6 months after therapy started. However, depending on the severity of the disease and on patient compliance with the experimental clinical protocol, sputum from some of them was collected at months 9, 10 and 11. For the same reasons, collection of the expectorate from members of the control group was performed at months 0, 2, 3, 4, 5, 6 and 7 after therapy began. To avoid sequencing batch effects, the sequencing libraries of all samples were pooled and sequenced together.

**Table 4:** Demographic characteristics of the subjects enrolled at the National Institute for Infectious Diseases in Rome, Italy.

		Pulmonary TB*	Pneumonia**	Total
Enrolled subjects (%)		10 (45)	12 (55)	22 (100)
**Median age in years (range)**		36 (25–60)	51 (28–74)	40 (25–74)
**Female gender (%)**		2 (20)	4 (33)	6 (27)
**Origin (%)**	Italy (%)	3 (30)	11 (92)	14 (64)
	Romania (%)	7 (70)	1 (8)	8 (36)
**BCG N (%)**	Vaccinated (%)	7 (70)	1 (8)	8 (36)
	Unvaccinated (%)	3 (30)	11 (92)	14 (64)
Antibiotic therapy before starting in-hospital therapy (%)***		6 (60)	10 (83)	16 (73)
**Smokers (%)**		9 (90)	3 (25)	12 (54)
	Mild Grade (%)	0	4 (33)	4 (18)
**Chest X-ray (%)**	Intermediate Grade (%)	1 (10)	6 (50)	7 (32)
	High Grade (%)	9 (90)	2 (17)	11 (50)
**Diabetes**		0	0	0

TB: Tuberculosis

*all TB cases were microbiologically confirmed; BCG: Bacillus Calmette–Guérin vaccine

**among the pneumonia patients, a subject affected by lung abscess was included

*** The therapy consisted of cephalosporins or macrolides. Sputum was collected within 7 days after therapy started (after 2 days on average). Information about BCG vaccination, smoking and diabetes was collected from clinical records.

**Fig 4:**
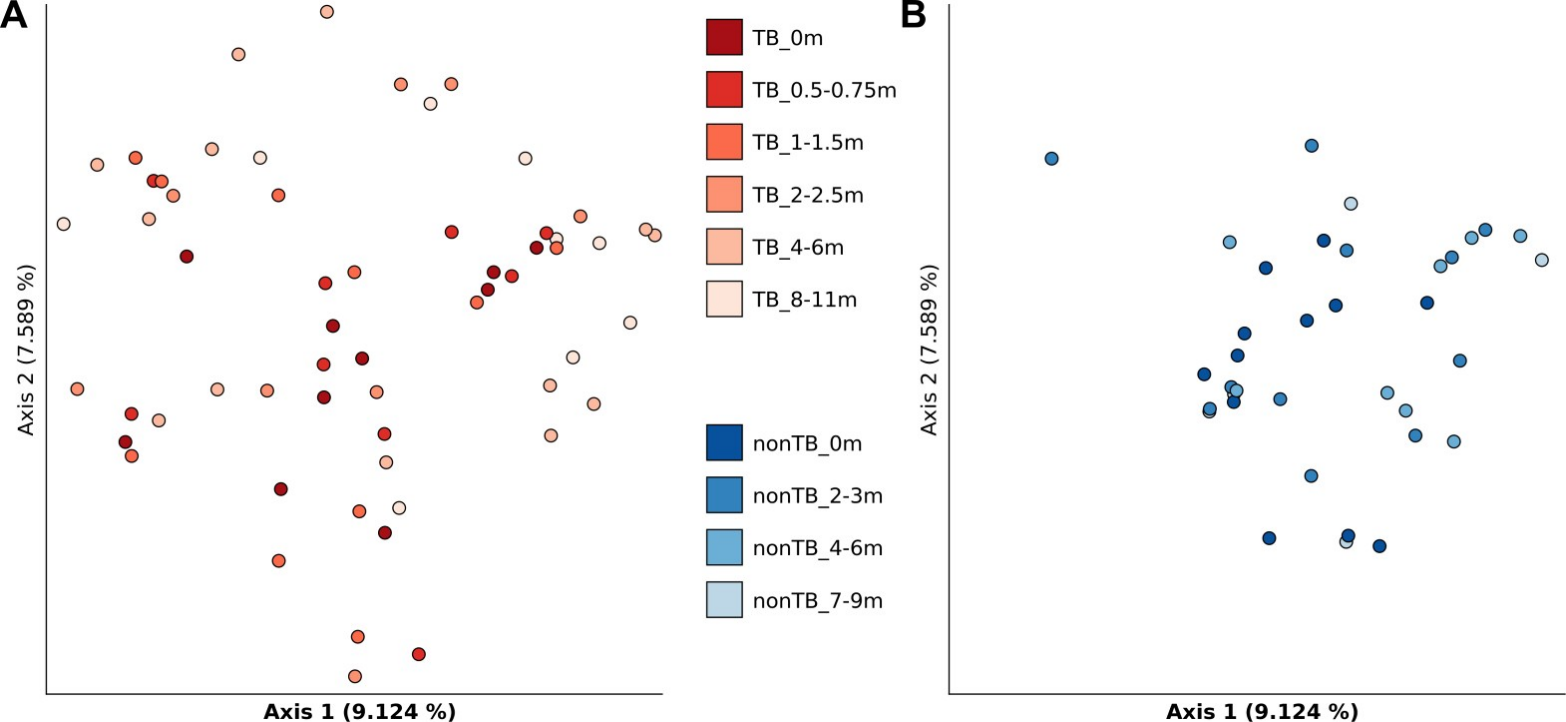
Principal component analysis (PCoA) of the sputum samples collected in Italy, based on Bray-Curtis distances. **A.** Samples collected from tuberculosis (TB) patients. The different colors indicate the various time-points. **B.** Samples collected from pneumonia (nonTB) patients. The different colors correspond to the various time-points.

Analysis of 16S rDNA Illumina reads proved that sequencing coverage was excellent for all of the samples, with more than 99.7% of the reads mapping to the Greengenes database [42]. S4 Fig shows the most abundant Phyla identified in the samples (Firmicutes, Bacteroidetes, Actinobacteria, Proteobacteria, Fusobacteria, TM7, Tenericutes, SR1 and Spirochaetes) and highlights the high variability observed. After normalization to sequencing depth and identification of the bacterial taxa represented in each sample, alpha diversity was calculated using the Faith Phylogenetic Diversity and the Shannon index. Overall, no correlation between time points was observed (Shannon: Pearson 0.1963, p-value 0.0566; Spearman 0.1807, p-value 0.0796; Faith: Pearson 0.2656, p-value 0.0093; Spearman 0.2228, p-value 0.0300), and the Kruskal-Wallis test revealed no significant difference between TB patients and patients with an unrelated lung disease (S5 Fig), and modest differences between the time-points (S6 Fig). Beta diversity calculation (i.e. quantitative measure of community dissimilarity) was calculated using the Bray-Curtis dissimilarity test [43] (based on abundances), the Jaccard index (based on the presence or absence of species) and unweighted and weighted UniFrac [44] methods (the latter incorporates phylogenetic relationships). PCoA did not reveal any clustering of either disease status or time-point for any of the methods mentioned above (Fig 4 shows the Bray-Curtis PCoA, PERMANOVA pseudo-F = 1.096, p-value = 0.137). No significant diversity between TB and non-TB patients was found for any of the beta diversity measures (PERMANOVA method, with 999 permutations: Jaccard, pseudo-F = 1.087, p-value = 0.051; Unweighted Unifrac, pseudo-F = 1.923, p-value = 0.001; Weighted Unifrac, pseudo-F = 1.711, p-value = 0.135).

Differential abundance analysis was obtained by using ANCOM (Analysis of composition of Microbiomes [45]) and the Gneiss method [46]. Statistical analysis for the ANCOM study was not significant. Upon analysis of the differential abundance of the various taxa, the Gneiss method proved that more than 70% of the community variation was due to variability among subjects and less than 20% could be attributable to other factors, such as sample groups or time-points (S7 Fig).

Finally, volatility analysis assessed how volatile a dependent variable was over a continuous, independent variable (i.e. time) in one or several groups. The Shannon alpha diversity index did not show any consistent time-dependent change in TB patients compared to non-TB patients, as illustrated in S8 Fig.

In summary, there were no differentially abundant features over time in the group of TB or control patients recruited in Italy.

### Microbial diversity in patients recruited in Bangladesh

The last group of sputum samples was collected at the “icddr,b institute” in Bangladesh, where 11 TB patients were recruited. These were sampled at different time-points throughout therapy, together with 10 healthy donors, who were sampled only once. Demographic and clinical features of the subjects enrolled are detailed in Table 5.

**Table 5:** Demographic characteristics of the subjects enrolled at the icddr,b, Dhaka, Bangladesh.

		Smear positive pulmonary TB patients	Healthy controls*	Total
Enrolled subjects (%)		11 (52)	10 (48)	21 (100)
**Median age in years (range)**		35 (24–50)	24 (23–25)	29 (23–50)
**Female gender (%)**		3 (27)	0 (0)	3 (14)
**Diabetes (%)**	Status known (%)	7 (64)	8 (80)	15 (71)
	Yes (%)	3 (43)	0	3 (20)
**Smokers (%)**		7 (64)	9 (90)	16 (76)
**Alcohol abusers (%)**		0	4 (40)	4 (19)
**Drug abusers (%)**		0	0	0

TB: Tuberculosis

*Healthy controls had no symptoms suggestive of TB during the period of enrolment. Only two controls were exposed to TB patients in their family. Information about smoking, alcoholism and drug abuse was collected from clinical records.

Technical issues (some sputa could not be collected, failure of library preparation or unsuccessful sequencing for other samples) excluded 2 healthy donors and 5 TB time-points from downstream analyses. Sequencing coverage was good in all of the remaining samples, with >99.5% of the reads mapping to the Greengenes [42] dataset.

**Fig 5:**
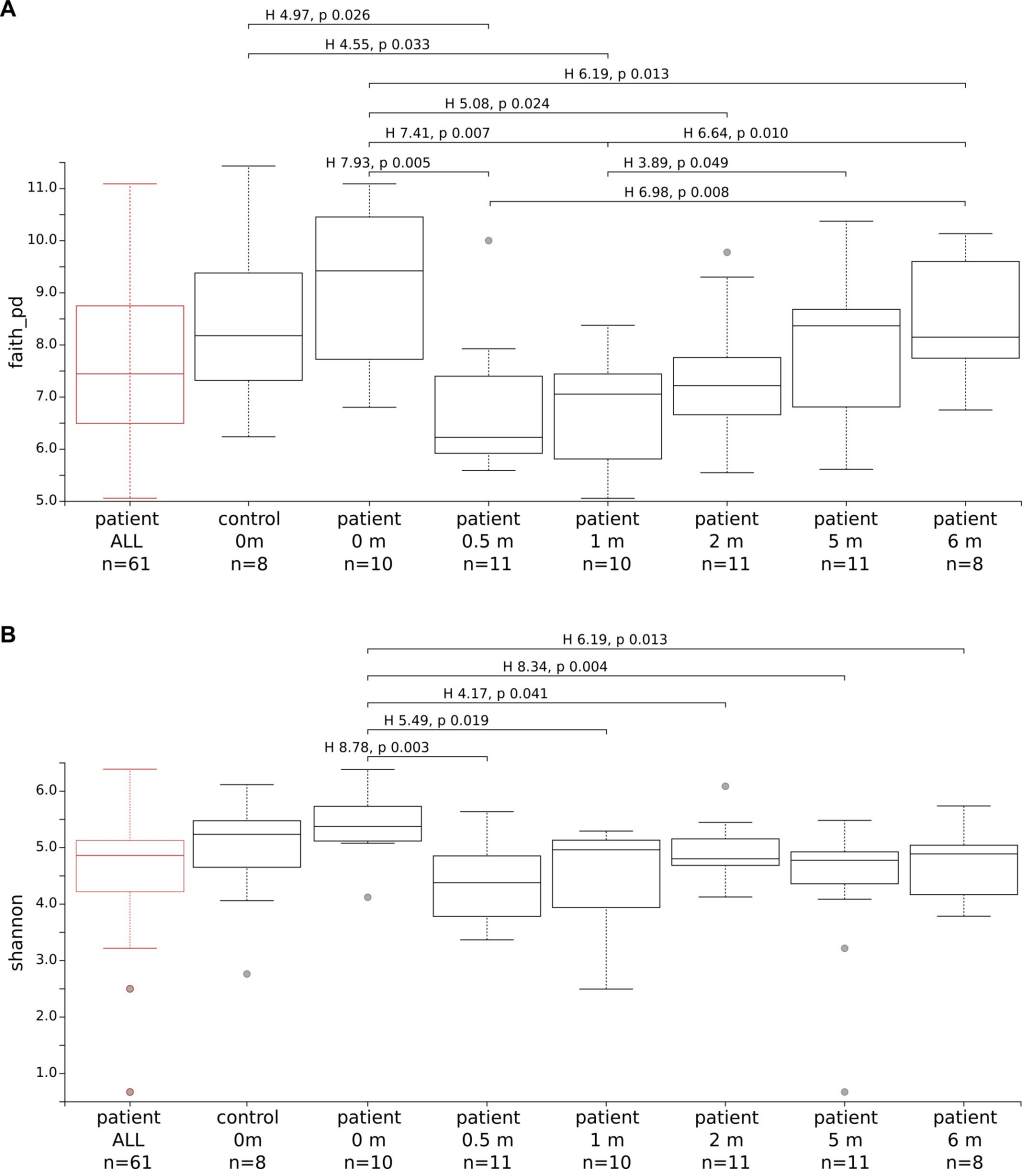
**Alpha diversity for the sputum samples received from Bangladesh. A.** Faith Phylogenetic Diversity. **B.** Shannon index. Samples from controls and TB patients are listed on the X-axis. TB patients are grouped according to the time-point. Grey dots represent outliers. Pairwise Kruskal-Wallis statistics are shown for p-values smaller than 0.05. The “patient ALL” group (red) was tested separately against the controls (test not statistically significant). Kruskal-Wallis test for all groups was 19.05 (p-value 0.004) for Faith Phylogenetic Diversity and 14.94 (p-value 0.021) for Shannon index.

**Fig 6:**
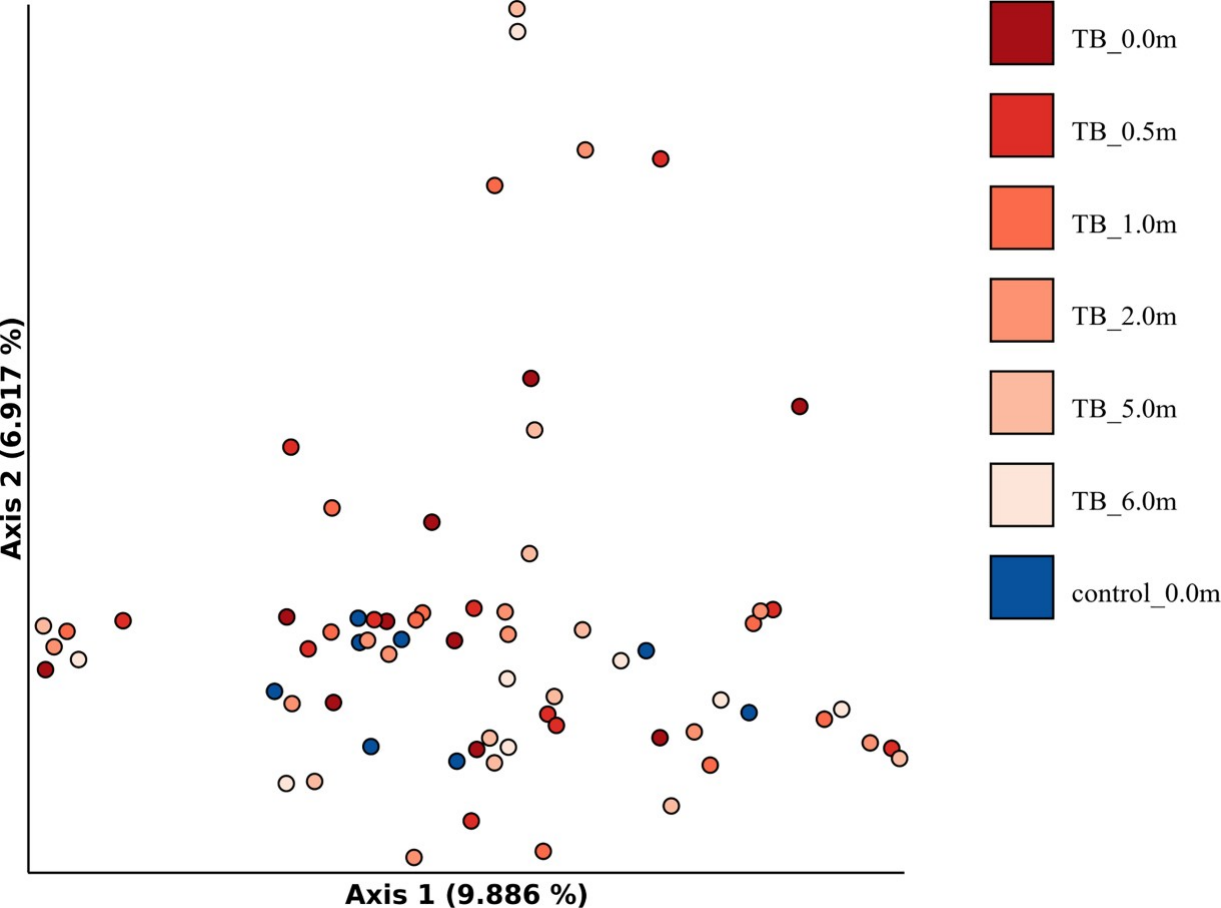
Principal component analysis (PCoA) of the sputum samples collected in Bangladesh. The analysis is based on Bray-Curtis distances and shows control and TB patients at the different time-points.

S9 Fig lists the most abundant Phyla identified in the samples, which overall matched those previously noticed in the sputa collected in Italy, that is: Firmicutes, Bacteroidetes, Actinobacteria, Proteobacteria, Fusobacteria, TM7, Tenericutes, SR1 and Spirochaetes. As reported for the Italian patients, high variability was observed among the Bangladeshi individuals as well. Alpha diversity was calculated for these samples as described above for the Italian cohort. No significant variation in alpha diversity was observed between the healthy donors and the TB group (Kruskal-Wallis: Faith Phylogenetic Diversity, H = 1.45, p-value = 0.2; Shannon index, H = 2,94, p-value = 0.09). In contrast to the Italian cohort, a modest decrease in alpha diversity was evident in TB patients after 2 weeks of anti-TB therapy as compared to healthy donor samples (Faith Phylogenetic Diversity, Kruskal-Wallis p-value = 0.026) and compared to TB samples at time-point 0 (Faith Phylogenetic Diversity, Kruskal-Wallis p-value = 0.005) (Fig 5). In addition, a PCoA analysis according to Bray-Curtis was performed as well to check whether samples clustered according to any metadata (i.e. control or TB, different time-points). Fig 6 shows that no clear grouping was possible. No significant difference between groups was found using the Jaccard index (PERMANOVA pseudo-F 1.317, p-value 0.056) and the unweighted and weighted UniFrac (PERMANOVA pseudo-F 1.115, p-value 0.32, and pseudo-F 3.272, p-value 0.035, respectively) (S10 Fig).

Differential abundance analysis was calculated by ANCOM [45] and Gneiss methods [46], as described for the samples collected in Italy. Significance for the ANCOM study was weak and not significant. Furthermore, Gneiss analysis of the differential abundance of the various taxa showed that most of the variability was due to variation between subjects and only about 5% was attributable to time (S11 Fig). Similarly to the observations made with samples obtained in Italy, no microbial pattern, i.e. no differentially abundant features, could be identified in either of the groups. Indeed, each patient was characterized by a specific microbiota profile, which did not allow extrapolation to a common signature.

Finally, volatility analysis evaluated variability of the alpha diversity over time. The Shannon index did not change during the 6-month timeframe in TB patients (S12 Fig). Therefore, results obtained with the cohort from Bangladesh confirmed the absence of features that are significantly and differentially abundant in people with active TB compared to those without the disease, at the time of diagnosis as well as over time.

## Discussion

Robust biomarkers that allow diagnosis of active, latent or re-activated TB disease and follow-up of TB patients during chemotherapy play a pivotal role in defining clinical interventions [47,48]. In addition to medical examination, a few biomarkers are nowadays available and comprise sputum conversion analysis, macrophage activation markers and detection of mycobacterial DNA, lipoarabinomannan or chemokines in urine samples [49–53]. Unfortunately, some of these biomarkers are of limited use in paucibacillary and pediatric TB, and of little or no use in latent TB, whereas others suffer from lack of consistency or limited applicability in specific settings. IFN-γ-release assays (IGRAs) such as QuantiFERON-TB Gold Plus and T-SPOT TB tests, measure responses to antigens (e.g. ESAT-6 or CFP-10) that discriminate infection from immunity induced by vaccination but not between active disease and latent *M*. *tuberculosis* infection [54].

The hypothesis that underpinned this study was that exploration of the sputum microbiota could identify and define a new class of microbiota-based biomarkers that might complement existing tools. To address this question, multicenter studies were undertaken to characterize sputum microbiota in the context of TB disease. Of these, three were independent prospective cohort studies. Results, based on 16S sequence analysis, revealed minor and inconsistent differences in microbial abundance in sputum samples.

No difference in alpha diversity was observed in TB or pneumonia patients. Only the Bangladeshi cohort showed a reduction of the alpha diversity 2 weeks after anti-TB therapy was initiated, and this then gradually increased with time. Interestingly, the microbial profiles of the people recruited in Dhaka seemed to be more homogeneous than those of the Italian cohort possibly because the latter showed more ethnic diversity.

Only sporadic, if any, differences were detected. Taxonomic composition varied greatly among patients and time points. This was especially obvious for the most prevalent species like *Streptococcus* and *Prevotella*, which in some samples accounted for the majority of the microbiome. Our analyses did not identify any particular taxon that was correlated with TB disease.

Previous studies of sputum microbiota were mainly conducted in China [29,30,32,34], Colombia [33] and India [31] thus making correlations with our study difficult. Moreover, most of the reports mentioned above were based on a single sample, collected before antibiotic treatment. Therefore, it is difficult to extrapolate any similarity or difference with the Italian and Bangladeshi group, which are characterized by a time-course analysis. On the other hand, a tentative comparison can be made with our FIND and CHUV cohorts. For instance, increased abundance of Neisseriaceae was noted in TB patients from the second FIND group, similarly to what was reported by Krishna and colleagues [31]. Bacteria belonging to the Bacteroidetes Phylum were found to be the most abundant in the CHUV set at time-point 0 and were also identified by Wu and colleagues [32] as among the most affected in their relative abundance studies.

The present work relies on collection of spontaneous or induced sputum samples, which may be contaminated by the microbial flora of the upper respiratory tract and therefore may not truly represent the microbial population in the lungs. Accurate lung sampling would require bronchoalveolar lavage but use of this rather invasive technique is unethical in a longitudinal study such as the one presented here.

This study is based on five datasets, each of which includes between 11 and 30 samples, split among TB and non-TB individuals. While these numbers fall within the order of magnitude of the sample size of previous works [30,31,33], the definition of a generic biomarker might require even bigger sample sets.

A possible limitation of this investigation was that *M*. *tuberculosis* was of seemingly low abundance as evidenced by 16S rDNA sequencing although the primers used for PCR successfully amplified the correct DNA fragment from purified genomic DNA. The reason for this is unknown although a study by Sulaiman and colleagues reported the same observation, i.e. the limitation of 16S rDNA amplification and sequencing in detecting mycobacteria in airway samples [55]. A more powerful approach for analysing the composition of sputum and lung microbiota could be metagenomics, possibly combined with transcriptomic and metabolomic analyses. While these procedures have been applied successfully to investigate the gut microbiota [56], the small amount of material that can be extracted from lung or sputum samples may limit their exploitation.

In conclusion, no association between the sputum microbiota composition and TB disease, or variation throughout anti-TB treatment, could be found in three different settings.

## Materials and methods

### Sample collection at the Foundation for Innovative New Diagnostics (FIND), Geneva, Switzerland

A request for sputum samples from HIV-negative TB and non-TB patients was submitted to The Foundation for Innovative New Diagnostics (FIND) by the laboratory of Prof. Cole at EPFL in November 2013 and then again in June 2014. The requests were reviewed by the FIND Specimen Bank Review Committee and approved. FIND donated thirty sputum samples originated from TB (15) and non-TB (15) individuals in November 2013. A second batch of samples, including 15 TB and 15 non-TB, was donated by FIND in June 2014. Non-TB individuals had been subjected to microbiological testing to rule out TB. Induced sputum samples were shipped frozen to EPFL for DNA preparation, 16S rDNA amplification, sequencing and bioinformatic analysis. Tables 1 and 2 show socio-demographic data of the patients whose samples were received from FIND.

### Study approval and sample collection at Centre Hospitalier Universitaire Vaudois (CHUV) in Lausanne, Switzerland

The study was approved by the Commission Cantonale d’étique de la Recherche sur l’être humain (CER-VD) with protocol 35/09. Written informed consent was obtained from ten HIV-negative patients diagnosed with culture-positive, pulmonary TB, who were enrolled in the study. Induced sputum samples were collected, decontaminated in (NALC/NaOH), stored at -80°C and shipped to EPFL for DNA preparation, 16S rDNA amplification, sequencing and bioinformatic analysis. Table 3 shows socio-demographic data of the subjects enrolled in Switzerland.

### Study approval and sample collection at the National Institute for Infectious Diseases (INMI) “L. Spallanzani” in Rome, Italy

The study was approved by the Ethics Committee of the National Institute for Infectious Diseases “L. Spallanzani” IRCCS with the “PARERE n. 98/2014” (October 20^th^, 2014) and all enrolled patients provided written informed consent. Twenty-two HIV-negative patients hospitalized for suspected pulmonary TB from November 2014 to July 2015 at “L. Spallanzani” Hospital for Infectious Diseases in Rome were enrolled. Among the 22 patients, 10 had culture-confirmed TB and 12 had pneumonia.

#### Patients with active TB

TB symptom screening was conducted according to internal guidelines of the National Institute for Infectious Diseases “L. Spallanzani” (Protocollo INMI di Gestione Clinica della Tubercolosi- Revisione N.7-Gennaio 2020 www.inmi.it/protocolli_e_linee_guida.html).

Culture-confirmed TB was defined as at least one positive culture for *M*. *tuberculosis* sensitive to first-line drugs from spontaneous or induced sputum. In particular, 8 patients were Acid Fast Bacilli (AFB) sputum smear positive and 2 AFB sputum smear negative; however, these 2 sputa were positive upon nucleic acid amplification (Xpert MTB/RIF assay; Cepheid, United States). Culture was performed on both solid media (proportion method in Lowenstein-Jensen medium) and liquid media (MGIT 960 systems; Becton Dickinson, Sparks, MD, USA).

Six TB patients out of 10 had been taking cephalosporins for maximum 2 days before being sampled. For treatment-naïve patients (4 out of 10), the first sputum sample was collected within 72 hours of the beginning of TB therapy. Initial treatment was provided on an in-hospital basis, until AFB sputum conversion (Xpert MTB/RIF assay) was achieved on three consecutive negative samples collected during one week. After discharge, patients were followed monthly on ambulatory care for the full course of treatment (6 months). Treatment outcome was successful (culture negative and asymptomatic patient) in all 10 TB patients.

#### Patients with pneumonia (non-TB)

Community-acquired pneumonia was defined as an acute infection of the pulmonary parenchyma that was associated with at least some symptoms of acute infection, accompanied by the presence of an acute infiltrate on a chest radiograph, in patients not hospitalized or residing in a long-term care facility for more than 14 days before onset of symptoms. All pneumonia patients were AFB sputum smear negative and culture negative for M. tuberculosis. A patient with lung abscess was also included in the group.

For both TB and pneumonia samples, from the second month of treatment onwards, all sputa were induced. All samples were decontaminated in N-acetyl-L-cysteine-sodium hydroxide (NALC/NaOH), stored at -80°C and shipped to EPFL for DNA preparation, 16S rDNA amplification, sequencing and bioinformatic analysis. Table 4 shows socio-demographic data of the Italian cohort.

### Study approval and sample collection at the icddr,b, Dhaka, Bangladesh

This study was approved by the Research Review Committee (RRC, 21 April 2014) and Ethical Review Committee (06 August 2014) of icddr,b. Patient enrolment started from 26 April 2014 just after receiving clearance from RRC. Newly registered smear positive pulmonary TB (PTB) patients were selected from Shyamoli Hospital, Dhaka on the basis of convenience to ensure follow up. Healthy volunteers (controls) were enrolled from the attendants of the patients visiting icddr,b Dhaka Hospital. All enrolled individuals gave written informed consent to participate in the study. The controls were free from any clinical symptom suggestive of TB at the time of enrolment and had no previous history of TB. TB symptom screening was conducted according to internal guidelines of the icddr,b, Dhaka. Sputum microbiological testing was performed to rule out TB in healthy controls.

#### Specimen collection

Upon informed consent, HIV-negative TB patients and controls were brought into an isolated room of both respective study sites for sample collection. Induced sputum specimens were collected from all the controls and from the PTB patients who were unable to cough a good quality sputum specimen especially during the later stages of treatment. Sputum specimens were collected with all aseptic precautions and by expert physicians/nurses only. The specimens were collected from the PTB patients at six time-points over the course of the 6-month anti-TB treatment: Day 0 (when the diagnosis is made, during enrolment), 2 weeks post-treatment initiation (1^st^ follow up), 4 weeks post-treatment initiation (2^nd^ follow up), 8 weeks post-treatment initiation (3^rd^ follow up), 5 months post-treatment initiation (4^th^ follow up) and 6 months post-treatment initiation (5^th^ follow up).

Regarding controls, induced sputum specimens were collected only during enrolment.

In the Bangladeshi cohort, four out of the 15 enrolled smear-positive PTB patients dropped out just after enrolment. Therefore, they were excluded from specimen analysis. Of the remaining 11 patients, two were lost to follow up at their last time point (end of treatment). We could complete all the follow-ups for the remaining 9 TB patients. On the other hand, 10 healthy controls were enrolled and single induced sputum from each of them was collected.

After collection, specimens were transported to the icddr,b Mycobacteriology Laboratory on the same working day. All the specimens were processed and decontaminated there according to standard NALC/NaOH method. The processed specimens were stored and shipped at -20°C to Ecole Polytechnique Fédérale de Lausanne (EPFL) in Switzerland for DNA preparation, 16S rDNA amplification, sequencing and bioinformatic analysis. A total of 74 sputum/induced sputum specimens from all study participants were collected. Table 5 shows socio-demographic data of 11 PTB patients and 10 controls.

### DNA extraction from sputum samples

Sputum samples were stored at -80°C until use. DNA was extracted from the sputum samples obtained in Italy and in Bangladesh with the QIAmp UCP Pathogen Mini kit (Qiagen), according to the manufacturer’s recommendations. Sputum samples obtained in Switzerland (CHUV and FIND) were processed with the MagNa Pure 96 System and MagNa Pure LC DNA Isolation kit (Roche), according to the protocols provided by the manufacturer. Purified DNA was quantified by Qubit fluorometer (Life Technologies). These manipulations were performed under Biosafety Level 3 (BSL3) containment.

### 16S rDNA amplification, library preparation and high-throughput sequencing

PCR amplification of two variable (V1-V2) regions (349 bp) of the 16S RNA gene was carried out using primers 5’-AGR GTT YGA TYM TGG CTC AG-3’ and 5’-TGC TGC CTC CCG TAG GAG T-3’ (Microsynth AG, Switzerland), where “R” could be A or G, “Y” could be C or T, “M” could be A or C [30]. Platinum PCR Super Mix High Fidelity (Life Technologies) was used, according to the manufacturer’s recommendations, with a no template sample as a negative control. PCR products were purified by MinElute PCR Purification Kit (Qiagen) and quantified using Qubit (Life Technologies). Sequencing libraries were prepared with the TruSeq ChIP Sample Prep Kit (Illumina) starting from 10 ng of PCR-amplified DNA and checked by Qubit and Fragment Analyzer (Agilent) before loading the sequencing cell. Multiplexed (S1 Table) high-throughput sequencing was performed at the Ecole Polytechnique Fédérale de Lausanne on a MiSeq instrument (Illumina), with MiSeq Reagent kit V2 500 cycles (Illumina), where libraries were diluted to 8 pM and pooled according to the origin of the sputum samples. PhiX (PhiX Control V3, Illumina) was spiked in (15%) to generate diversity in the sequencing clusters.

### Bioinformatic analysis

Please refer to S1 Table for sequencing statistics. The sets of samples from FIND and the sputum samples received from CHUV were analysed as follows. Sequences were quality trimmed with Trimmomatic [57] and paired reads were merged with SeqPrep [58]. Sequencing reads were mapped against Greengenes [42] v13.5 using bowtie2 [59], with the following parameters: -k 50 (50 alignment matches), -N 1 (only 1 mismatch), -L 20 (seed length of 20 nt),—end-to-end -p 5. For each Greengene ID, the counts were obtained by summing up the number of reads mapped to the genes, corrected for multiple hits (reads with multiple hits *mh* were counted as 1/*mh*). Bacteria represented in less than 10 samples were filtered out. Raw counts were then normalized prior to further analysis to modified z-scores, that is log counts were normalized to the sample medians and to the Median Absolute Deviation (MAD). Statistical analysis was obtained in R (version 3.3.1, https://www.r-project.org/) using standard packages (e.g. stats, base, utils) as well as limma (v. 3.28, https://www.bioconductor.org/packages/release/bioc/html/limma.html) for differential abundance analysis, and ggplot (v. 2.3, https://ggplot2.tidyverse.org/reference/). PCoA plots were obtained by applying the prcomp method (R package stats) on centered and scaled normalized abundances. Clusters were obtained from normalized and scaled abundances, running a hierarchical clustering (using the hclust method from the R package stats) based on a Pearson correlation distance. Heatmaps were plotted using the R package pheatmap (v.1.0). Differential abundance analyses were performed using the R package limma (v. 3.28). Significant bacteria were selected using an absolute log2 Fold-change of 1 and an adjusted p-value (FDR) of 0.05.

The samples from Italy and Bangladesh were analysed in the QIIME2 package (v. 2018.8) [60], using Greengenes v13.8 as the reference database. Greengenes 97% similarity set was used. OTUs were picked using the default parameters and the “—enable_rev_strand_match” option. Data was rarefied for even depth after reviewing the OUT table. The quality filter was set to 30 (quality-filter module) and deblur was used for denoising the reads. Sklearn (https://scikit-learn.org/stable/) was used for the taxonomy classification. The Naive Bayes classifier was trained on the targeted 16S region of the 99_otus Greengenes dataset (extracted using the primer sequences (see above) and the feature-classifier extract-reads module).

## Supporting information

S1 Fig Alpha diversity of the first (A) and second (B) batch of sputum samples received from FIND. The Shannon index was calculated at the Phylum, Class, Order, Family and Genus levels. TB samples are in violet, non-TB samples are in green. p-values of individual two-sided t-tests are shown.
(PDF)

S2 Fig Analysis of the second batch of samples received from FIND. A. PCoA (PC1 vs. PC2) displaying the samples, colored by sequencing batch, shaped by TB and filled by country. The total variance explained for the first components is shown in the inset. PCoA scores are shown in the boxplots below the graph. Segregation of TB and non-TB samples was noted on PC1. B. PCoA (PC1 vs. PC2) on bacteria, colored by Phylum. Bacteroidetes and Fusobacteria tend to segregate on PC1, while Firmicutes and Proteobacteria segregate on PC2. The boxplots show PC1 and PC2 scores per Phylum.
(PDF)

S3 Fig Heatmaps of mean (top) and median (bottom) abundances for samples received from CHUV. Hierarchical clustering was applied to all taxonomical levels (Phylum, Class, Order, Family, Genus and Species). Samples are ordered by time-point.
(PDF)

S4 Fig Taxonomy of the sputum samples received from Italy at the Phylum level. Samples from TB and non-TB patients are listed on the X-axis and grouped according to the time-point. The relative abundance of each Phylum is indicated on the Y-axis.
(PDF)

S5 Fig Alpha diversity (Faith Phylogenetic Diversity in A and Shannon index in B) for the sputum samples received from Italy. Samples from TB and non-TB patients were grouped and are indicated on the X-axis. Results of Kruskal-Wallis test are reported in the figure. p-value thresholding: 0.01.
(PDF)

S6 Fig Alpha diversity for the sputum samples received from Italy. **A.** Faith Phylogenetic Diversity. **B.** Shannon index. Samples from TB and non-TB patients, grouped according to the time-point, are listed on the X-axis. Grey dots represent outliers. Pairwise Kruskal-Wallis statistics are shown for p-values smaller than 0.05. Kruskal-Wallis test for all groups was 13.9 (p-value 0.126) for the Faith Phylogenetic Diversity and 13.89 (p-value 0.123) for the Shannon’s index.
(PDF)

S7 Fig Gneiss analysis to infer features that are differentially abundant in sputum samples received from Italy. The figure shows a dendrogram heatmap of relative abundances between sets of taxa (balances). Individual samples likely contributed to the most prominent balances.
(PDF)

S8 Fig Volatility analysis for the sputum samples received from Italy measured by the Shannon index. Continuous black bar: global mean. Error bars are shown. Global control limits (+/- 2x and 3x standard deviations from global mean) are indicated by dotted bars.
(PDF)

S9 Fig Taxonomy at the Phylum level of the sputum samples received from Bangladesh. Samples from controls or TB patients are listed on the X-axis and grouped according to the time-point. The relative abundance of each Phylum is indicated on the Y-axis.
(PDF)

S10 Fig Principal component analysis (PCoA) of the sputum samples collected in Bangladesh, based on the Jaccard (A) and Unifrac (B) distances. No segregation was noted.
(PDF)

S11 Fig Gneiss analysis to infer features that are differentially abundant in sputum samples received from Bangladesh. The figure shows a dendrogram heatmap of relative abundances between sets of taxa (balances). Individual samples likely contributed to the most prominent balances.
(PDF)

S12 Fig Volatility analysis for the sputum samples received from Bangladesh measured by the Shannon index. Continuous black bar: global mean. Error bars are shown. Global control limits (+/- 2x and 3x standard deviations from global mean) are indicated by dotted bars.
(PDF)

S1 Table Sequencing statistics. (XLSX)
