# Supplementary material for: Multicenter analysis of sputum microbiota in tuberculosis patients
Source: PLoS One. 2020 Oct 12;15(10):e0240250. doi: 10.1371/journal.pone.0240250 (PMC7549818; doi:10.1371/journal.pone.0240250)
Supplement: S1 Fig — Alpha diversity of the first (A) and second (B) batch of sputum samples received from FIND. The Shannon index was calculated at the Phylum, Class, Order, Family and Genus levels. TB samples are in violet, non-TB samples are in green. p-values of individual two-sided t-tests are shown. (PDF) [file pone.0240250.s001.pdf]

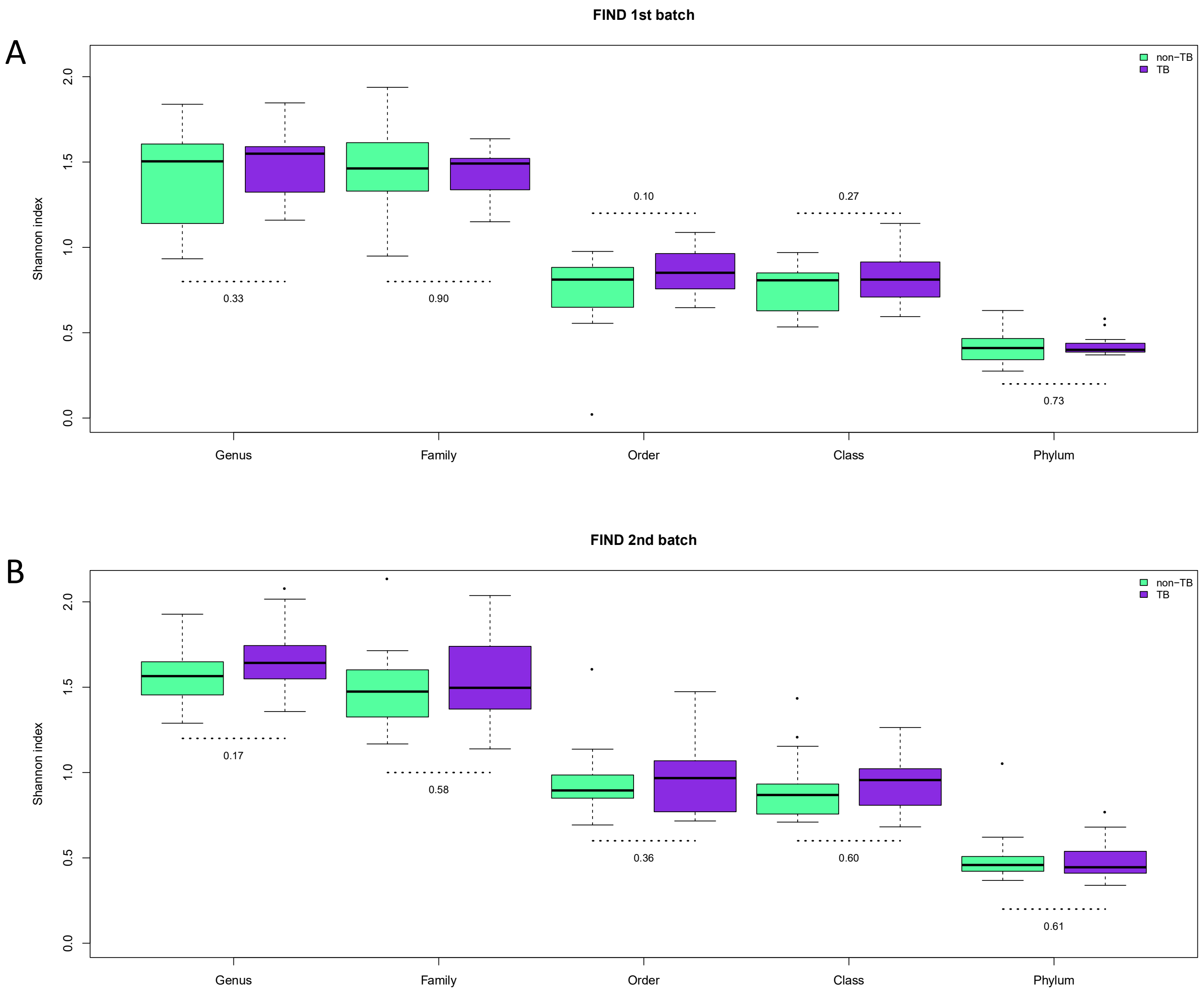

S1 Figure. Alpha diversity of the first (A) and second (B) batch of sputum samples received from FIND. The Shannon index was calculated at the Phylum, Class, Order, Family and Genus levels. TB samples are in violet, non-TB samples are in green, p-values of individual two-sided t-tests are shown.
