# Supplementary material for: Multicenter analysis of sputum microbiota in tuberculosis patients
Source: PLoS One. 2020 Oct 12;15(10):e0240250. doi: 10.1371/journal.pone.0240250 (PMC7549818; doi:10.1371/journal.pone.0240250)
Supplement: S2 Fig — A. PCoA (PC1 vs. PC2) displaying the samples, colored by sequencing batch, shaped by TB and filled by country. The total variance explained for the first components is shown in the inset. PCoA scores are shown in the boxplots below the graph. Segregation of TB and non-TB samples was noted on PC1. B. PCoA (PC1 vs. PC2) on bacteria, colored by Phylum. Bacteroidetes and Fusobacteria tend to segregate on PC1, while Firmicutes and Proteobacteria segregate on PC2. The boxplots show PC1 and PC2 scores per Phylum. (PDF) [file pone.0240250.s002.pdf]

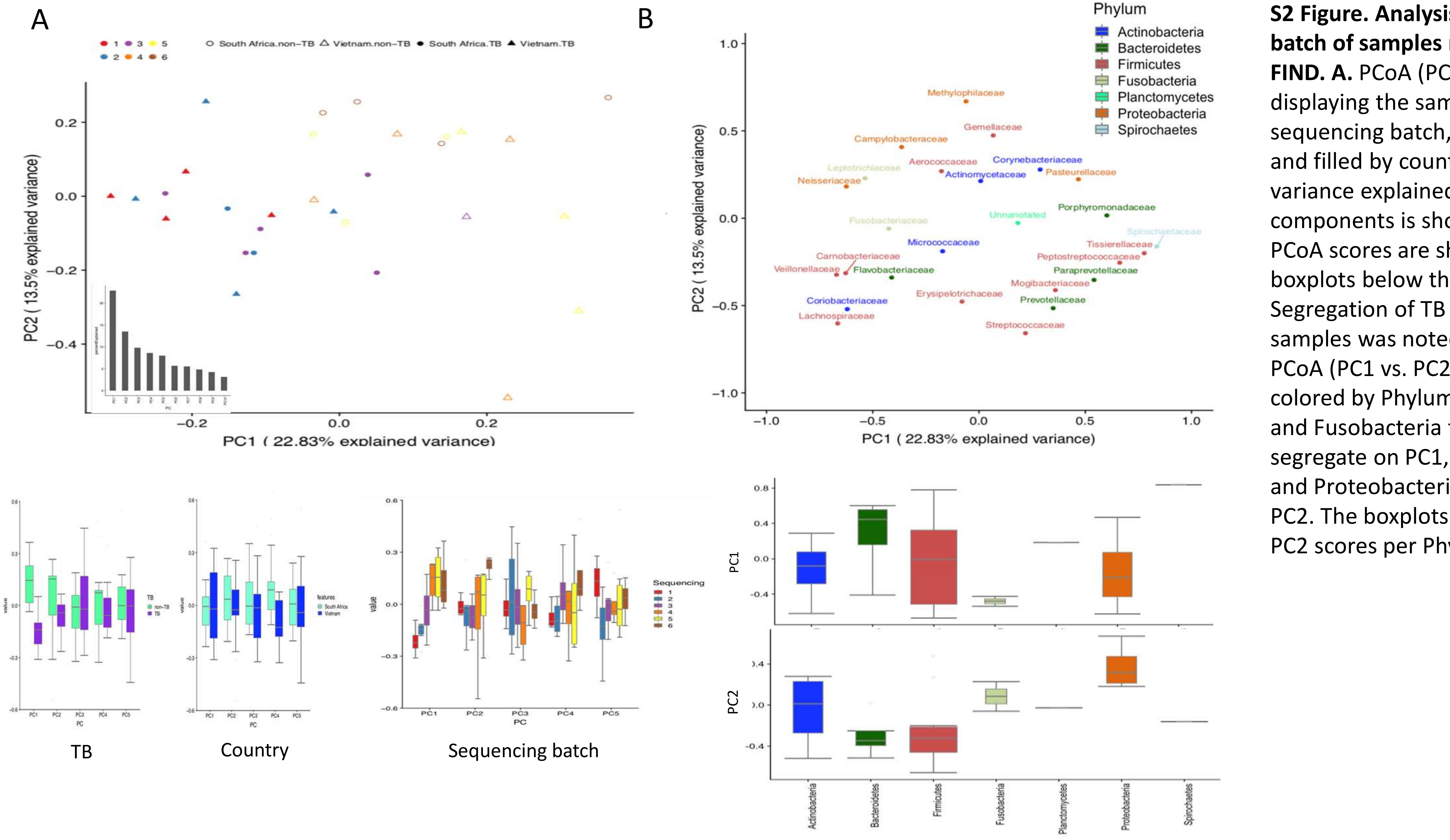


**S2 Figure. Analysis of the second batch of samples received from FIND. A.** PCoA (PC1 vs. PC2) displaying the samples, colored by sequencing batch, shaped by TB and filled by country. The total variance explained for the first components is shown in the inset. PCoA scores are shown in the boxplots below the graph. Segregation of TB and non-TB samples was noted on PC1. **B.** PCoA (PC1 vs. PC2) on bacteria, colored by Phylum. Bacteroidetes and Fusobacteria tend to segregate on PC1, while Firmicutes and Proteobacteria segregate on PC2. The boxplots show PC1 and PC2 scores per Phylum.
