# Supplementary material for: Multicenter analysis of sputum microbiota in tuberculosis patients
Source: PLoS One. 2020 Oct 12;15(10):e0240250. doi: 10.1371/journal.pone.0240250 (PMC7549818; doi:10.1371/journal.pone.0240250)
Supplement: S3 Fig — Heatmaps of mean (top) and median (bottom) abundances for samples received from CHUV. Hierarchical clustering was applied to all taxonomical levels (Phylum, Class, Order, Family, Genus and Species). Samples are ordered by time-point. (PDF) [file pone.0240250.s003.pdf]

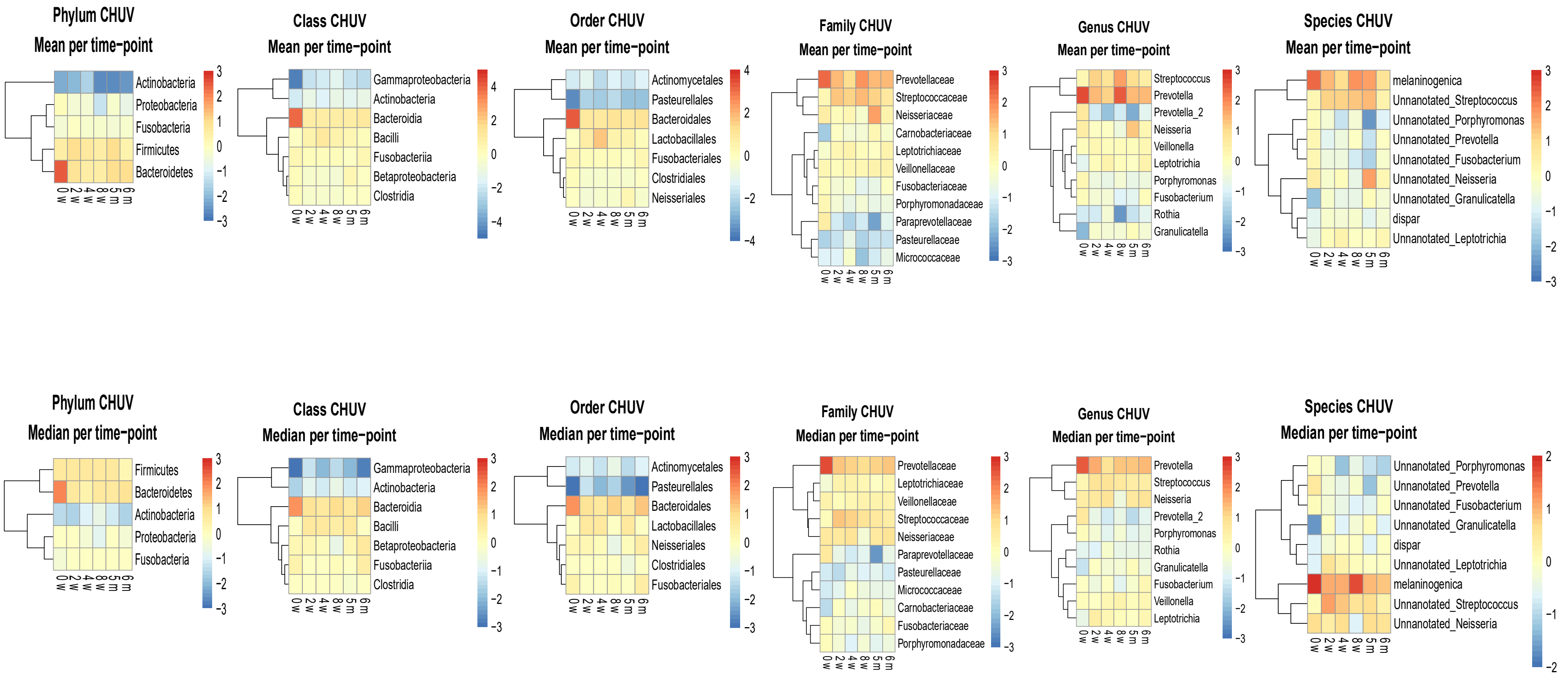

S3 Figure. Heatmaps of mean (top) and median (bottom) abundances for samples received from CHUV. Hierarchical clustering was applied to all taxonomical levels (Phylum, Class, Order, Family, Genus and Species). Samples are ordered by time-point.
