# Supplementary material for: Multicenter analysis of sputum microbiota in tuberculosis patients
Source: PLoS One. 2020 Oct 12;15(10):e0240250. doi: 10.1371/journal.pone.0240250 (PMC7549818; doi:10.1371/journal.pone.0240250)
Supplement: S4 Fig — Samples from TB and non-TB patients are listed on the X-axis and grouped according to the time-point. The relative abundance of each Phylum is indicated on the Y-axis. (PDF) [file pone.0240250.s004.pdf]

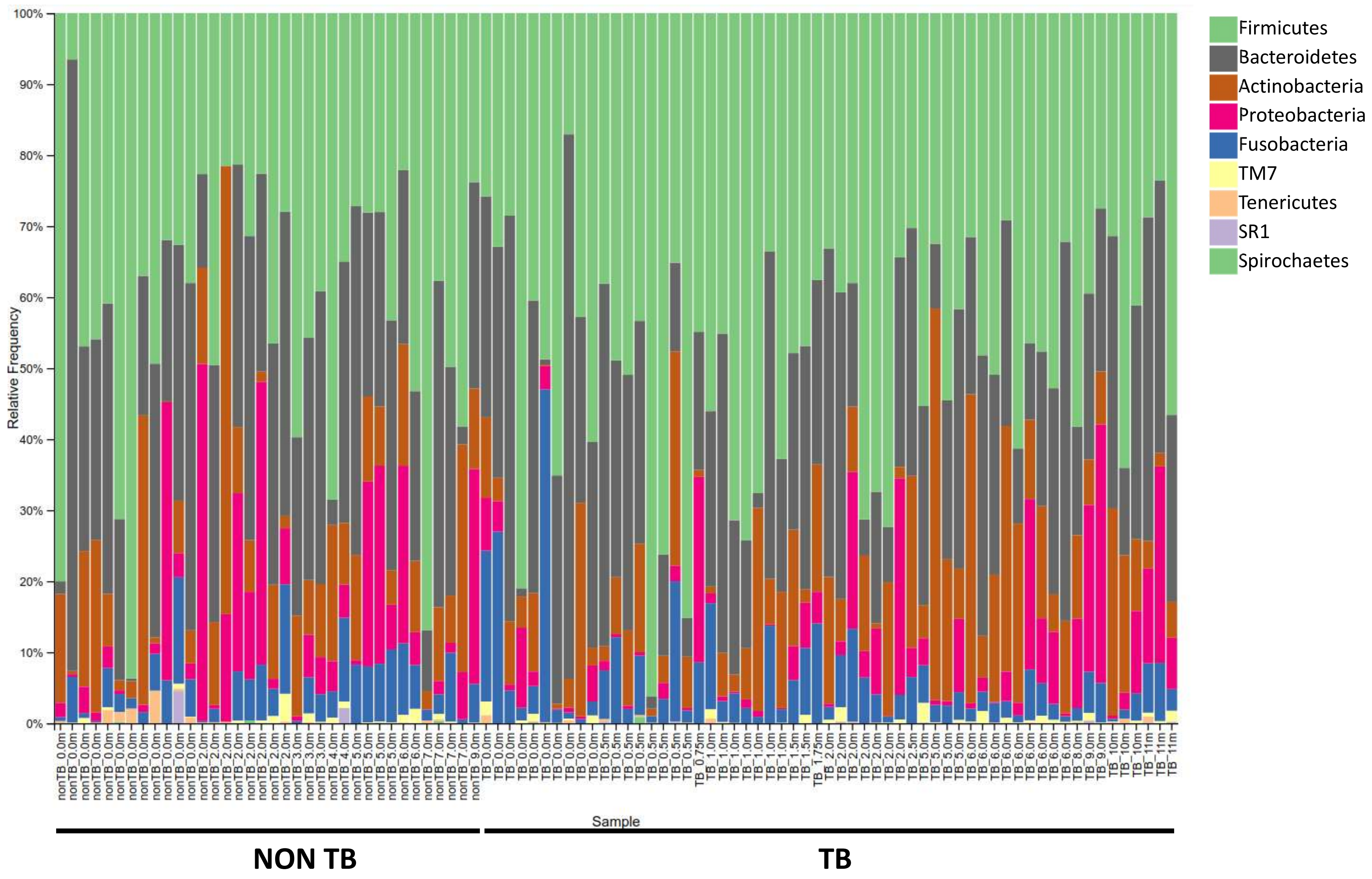

S4 Figure. Taxonomy of the sputum samples received from Italy at the Phylum level. Samples from TB and non-TB patients are listed on the X-axis and grouped according to the time-point. The relative abundance of each Phylum is indicated on the Y-axis.
