# Supplementary material for: Multicenter analysis of sputum microbiota in tuberculosis patients
Source: PLoS One. 2020 Oct 12;15(10):e0240250. doi: 10.1371/journal.pone.0240250 (PMC7549818; doi:10.1371/journal.pone.0240250)
Supplement: S5 Fig — Alpha diversity (Faith Phylogenetic Diversity in A and Shannon index in B) for the sputum samples received from Italy. Samples from TB and non-TB patients were grouped and are indicated on the X-axis. Results of Kruskal-Wallis test are reported in the figure. p-value thresholding: 0.01. (PDF) [file pone.0240250.s005.pdf]

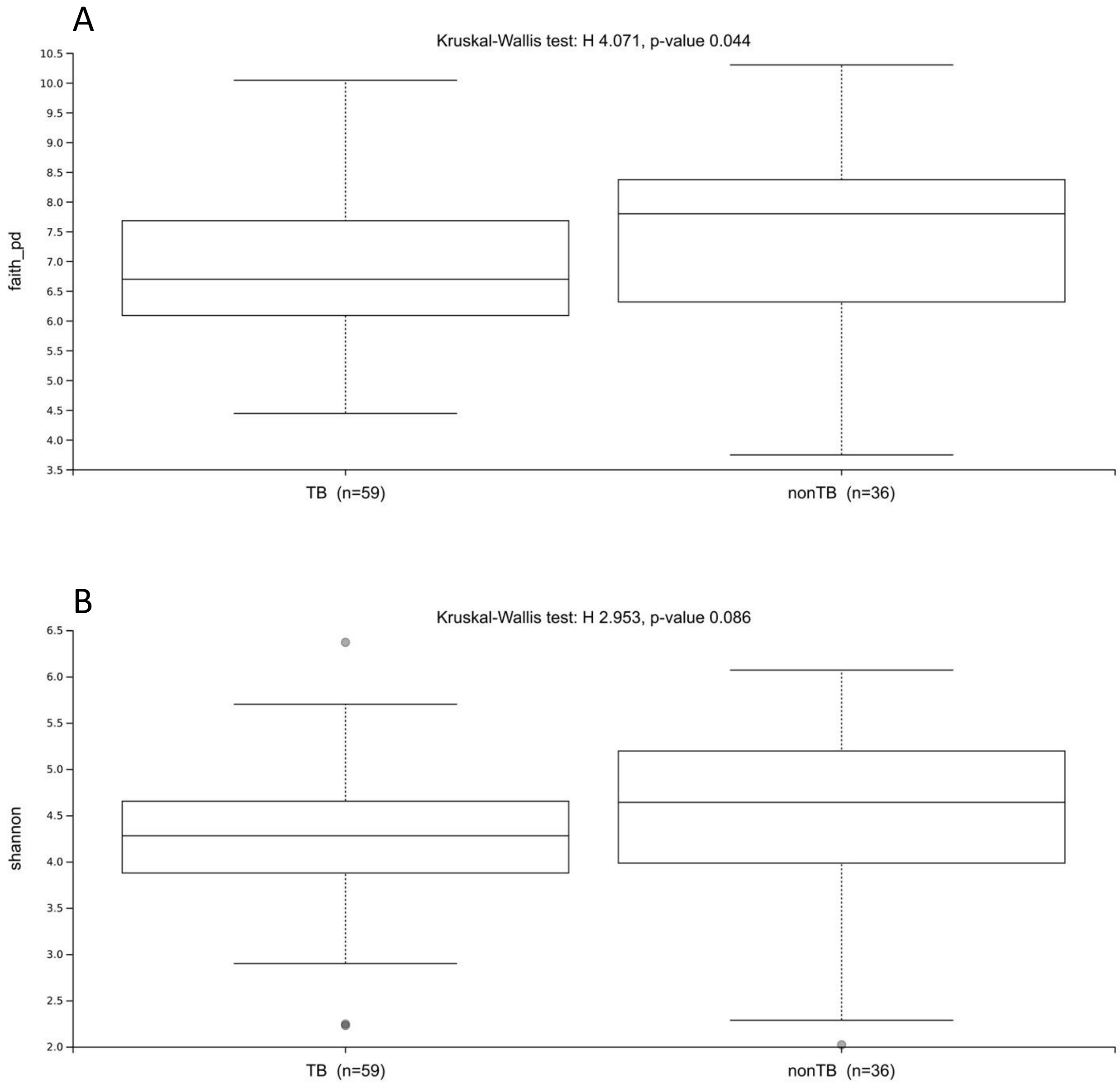

S5 Figure. Alpha diversity (Faith Phylogenetic Diversity in A and Shannon index in B) for the sputum samples received from Italy. Samples from TB and non-TB patients were grouped and are indicated on the X-axis. Results of Kruskal-Wallis test are reported in the figure. p-value thresholding: 0.01.
