# Supplementary material for: Multicenter analysis of sputum microbiota in tuberculosis patients
Source: PLoS One. 2020 Oct 12;15(10):e0240250. doi: 10.1371/journal.pone.0240250 (PMC7549818; doi:10.1371/journal.pone.0240250)
Supplement: S6 Fig — A. Faith Phylogenetic Diversity. B. Shannon index. Samples from TB and non-TB patients, grouped according to the time-point, are listed on the X-axis. Grey dots represent outliers. Pairwise Kruskal-Wallis statistics are shown for p-values smaller than 0.05. Kruskal-Wallis test for all groups was 13.9 (p-value 0.126) for the Faith Phylogenetic Diversity and 13.89 (p-value 0.123) for the Shannon’s index. (PDF) [file pone.0240250.s006.pdf]

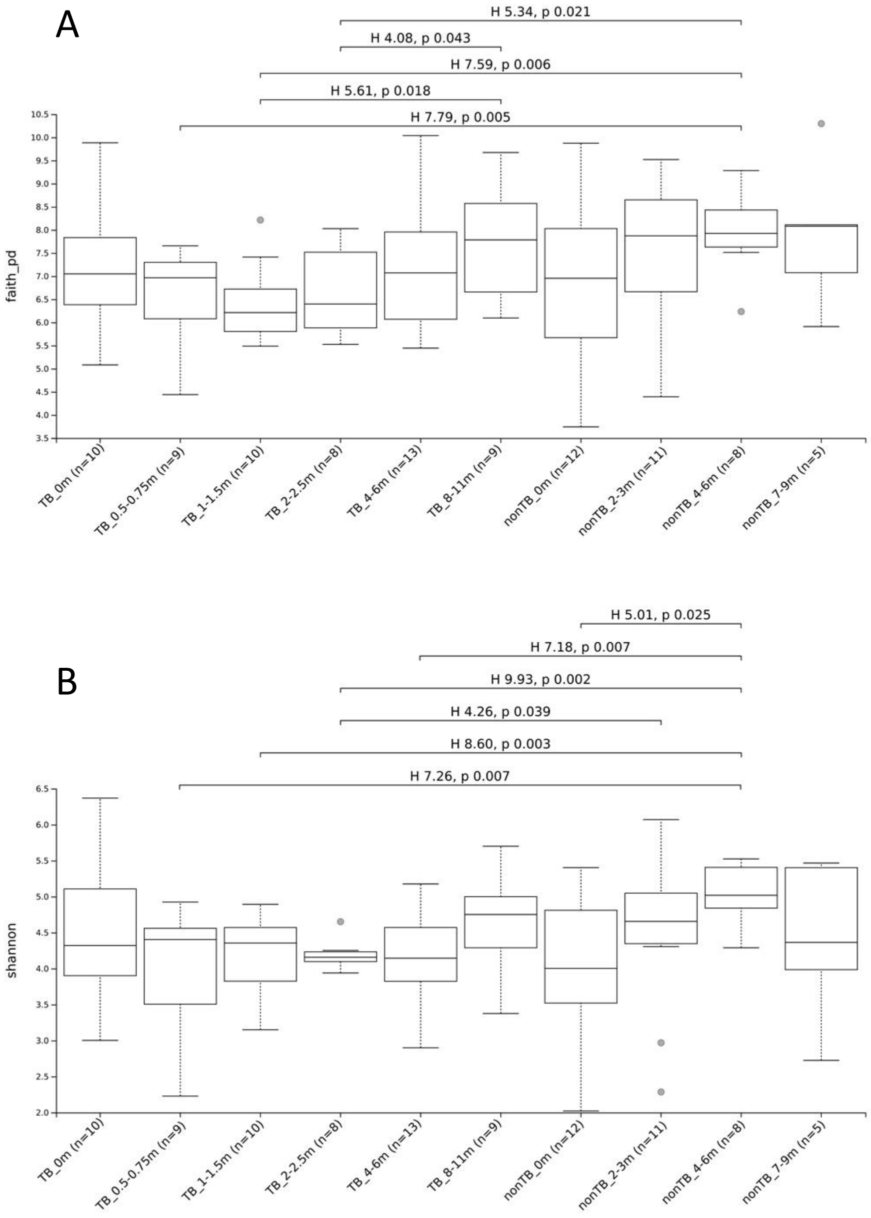

S6 Figure. Alpha diversity for the sputum samples received from Italy. A. Faith Phylogenetic Diversity. **B.** Shannon index. Samples from TB and non-TB patients, grouped according to the time-point, are listed on the X-axis. Grey dots represent outliers. Pairwise Kruskal-Wallis statistics are shown for p-values smaller than 0.05. Kruskal-Wallis test for all groups was 13.9 (p-value 0.126) for the Faith Phylogenetic Diversity and 13.89 (p-value 0.123) for the Shannon index.
