# Supplementary material for: Multicenter analysis of sputum microbiota in tuberculosis patients
Source: PLoS One. 2020 Oct 12;15(10):e0240250. doi: 10.1371/journal.pone.0240250 (PMC7549818; doi:10.1371/journal.pone.0240250)
Supplement: S9 Fig — Samples from controls or TB patients are listed on the X-axis and grouped according to the time-point. The relative abundance of each Phylum is indicated on the Y-axis. (PDF) [file pone.0240250.s009.pdf]

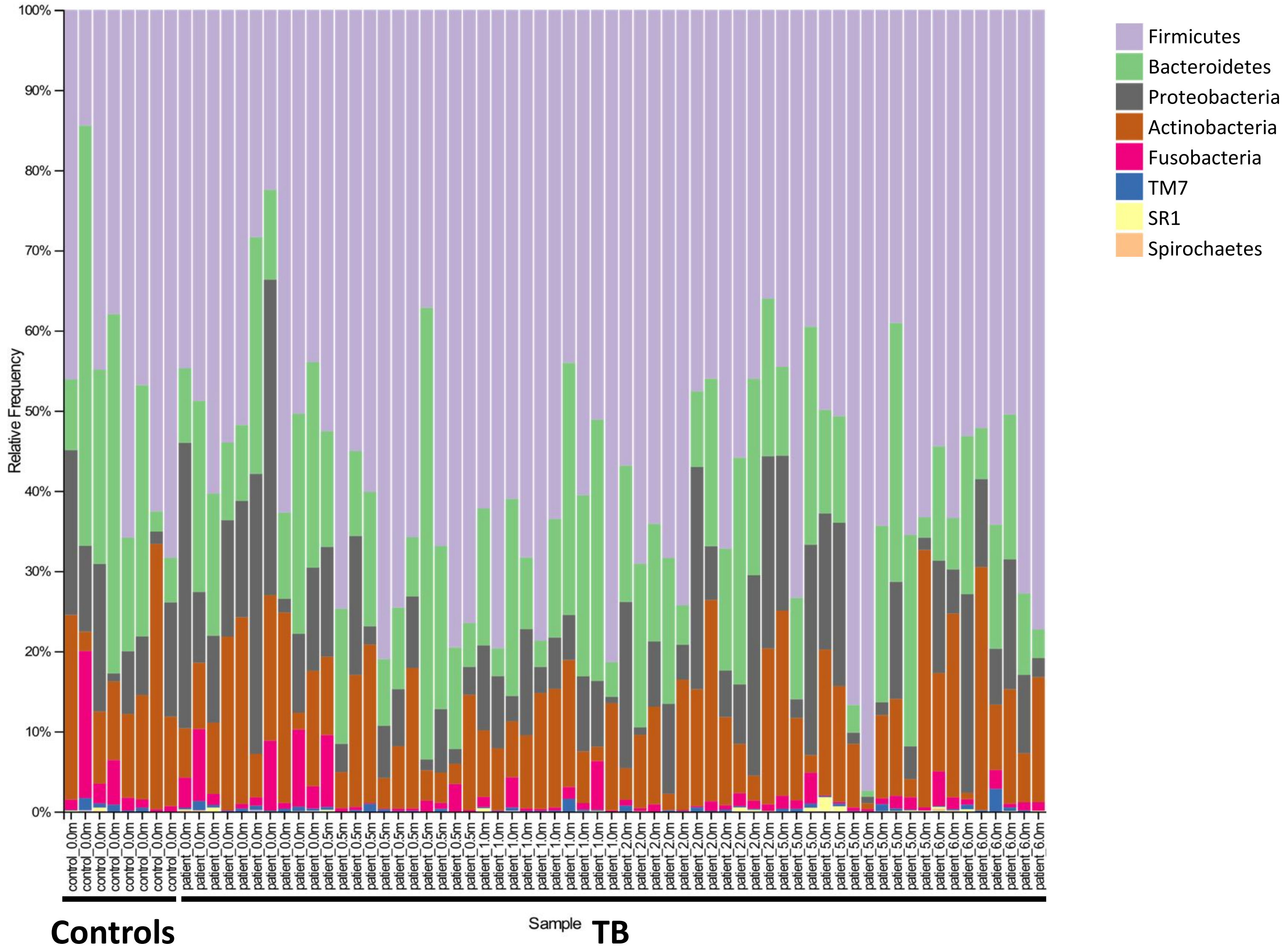

S9 Figure. Taxonomy at the Phylum level of the sputum samples received from Bangladesh. Samples from controls or TB patients are listed on the X-axis and grouped according to the time-point. The relative abundance of each Phylum is indicated on the Y-axis.
