# Supplementary material for: Multicenter analysis of sputum microbiota in tuberculosis patients
Source: PLoS One. 2020 Oct 12;15(10):e0240250. doi: 10.1371/journal.pone.0240250 (PMC7549818; doi:10.1371/journal.pone.0240250)
Supplement: S10 Fig — Principal component analysis (PCoA) of the sputum samples collected in Bangladesh, based on the Jaccard (A) and Unifrac (B) distances. No segregation was noted. (PDF) [file pone.0240250.s010.pdf]

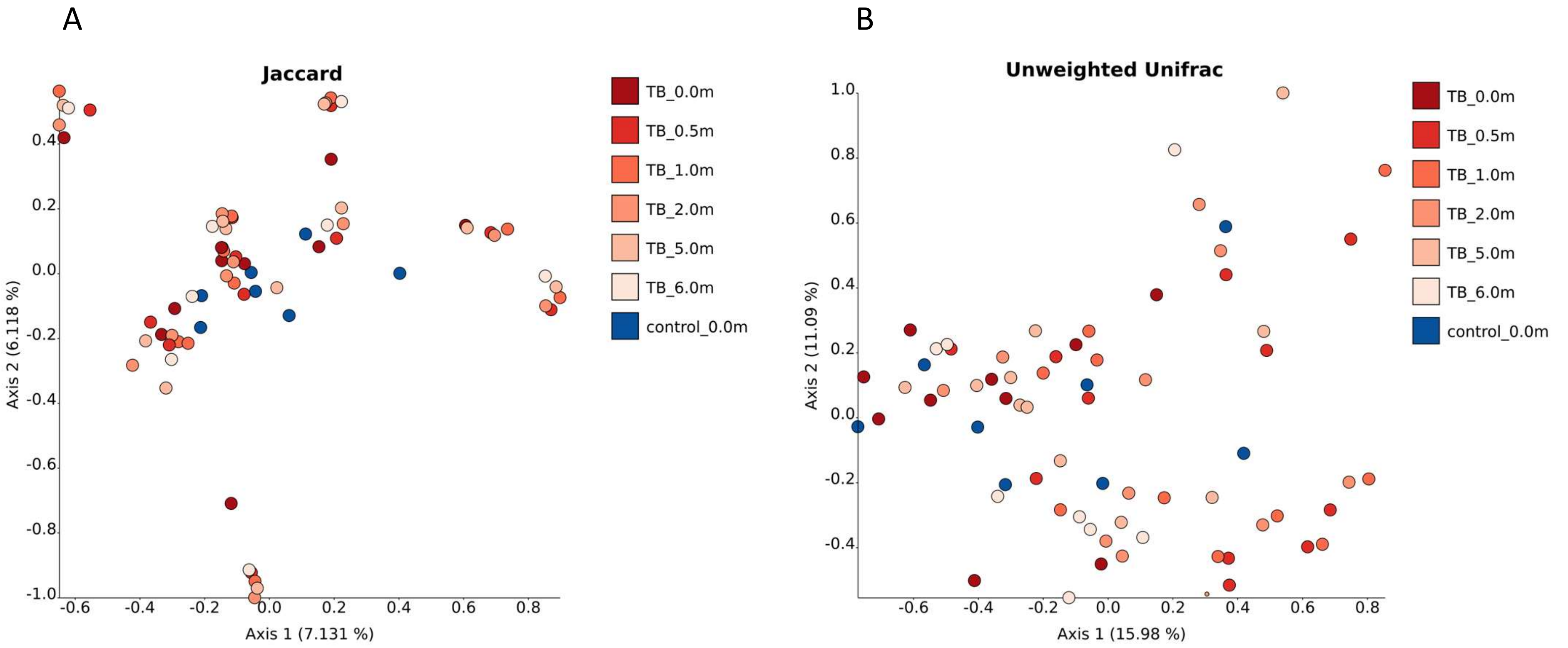

S10 Figure. Principal component analysis (PCoA) of the sputum samples collected in Bangladesh, based on the Jaccard (A) and Unifrac (B) distances. No segregation was noted.
