# Supplementary material for: Multicenter analysis of sputum microbiota in tuberculosis patients
Source: PLoS One. 2020 Oct 12;15(10):e0240250. doi: 10.1371/journal.pone.0240250 (PMC7549818; doi:10.1371/journal.pone.0240250)
Supplement: S11 Fig — The figure shows a dendrogram heatmap of relative abundances between sets of taxa (balances). Individual samples likely contributed to the most prominent balances. (PDF) [file pone.0240250.s011.pdf]

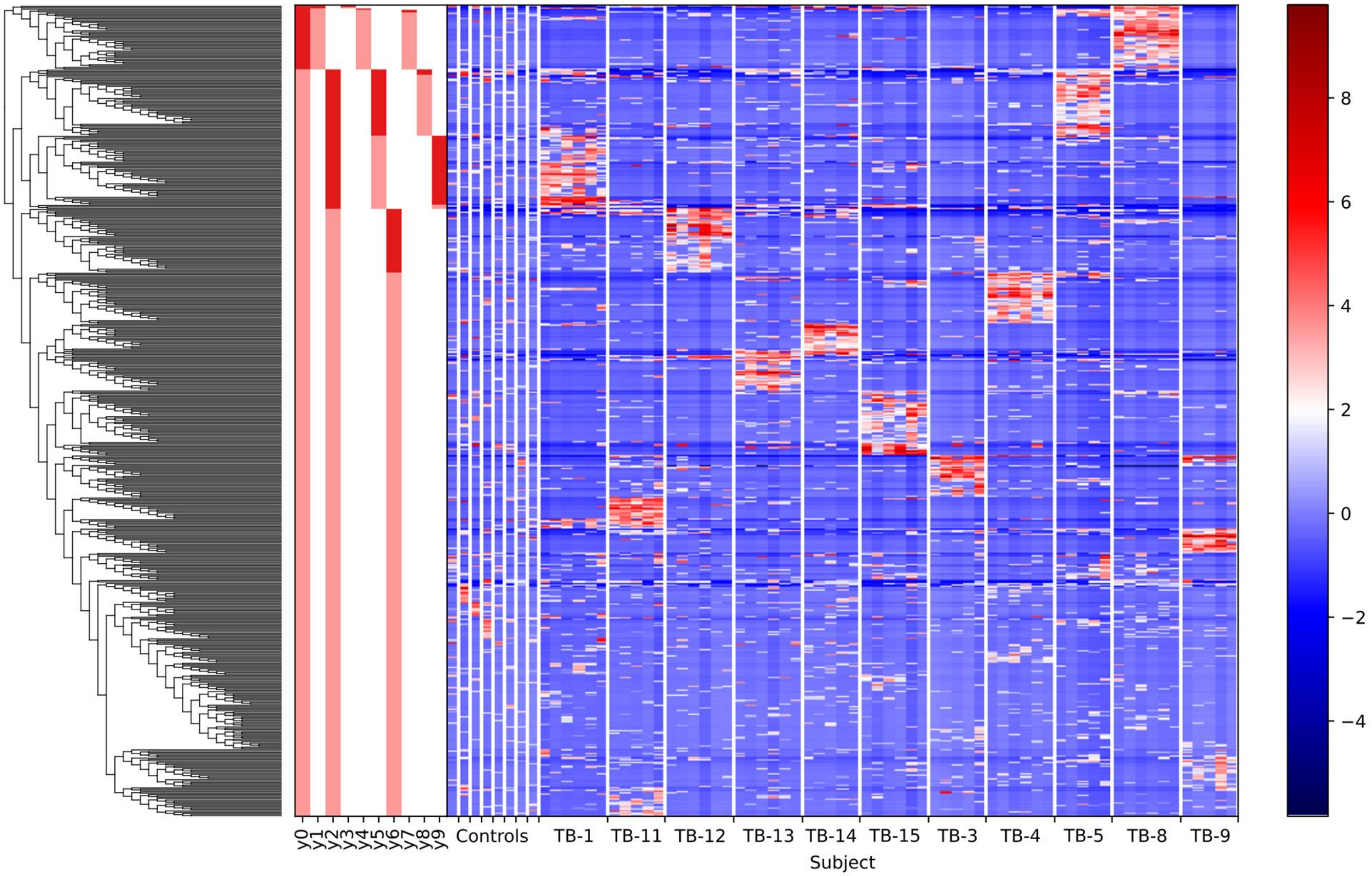

S11 Figure. Gneiss analysis to infer features that are differentially abundant in sputum samples received from Bangladesh. The figure shows a dendrogram heatmap of relative abundances between sets of taxa (balances). Individual samples likely contributed to the most prominent balances.
