# Supplementary material for: Multicenter analysis of sputum microbiota in tuberculosis patients
Source: PLoS One. 2020 Oct 12;15(10):e0240250. doi: 10.1371/journal.pone.0240250 (PMC7549818; doi:10.1371/journal.pone.0240250)
Supplement: S12 Fig — Continuous black bar: global mean. Error bars are shown. Global control limits (+/- 2x and 3x standard deviations from global mean) are indicated by dotted bars. (PDF) [file pone.0240250.s012.pdf]

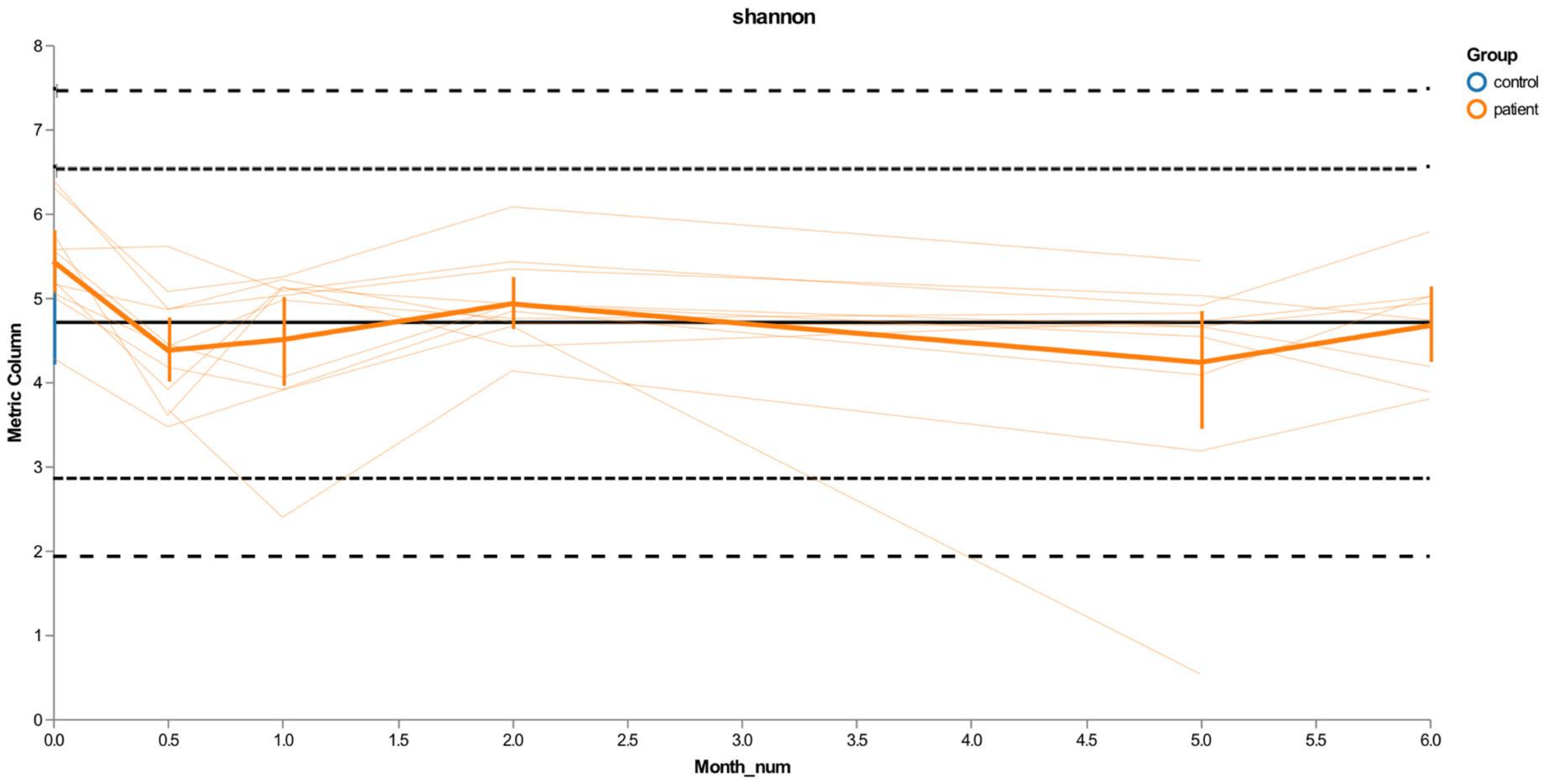

S12 Figure. Volatility analysis for the sputum samples received from Bangladesh measured by the Shannon index. Continuous black bar: global mean. Error bars are shown. Global control limits (+/- 2x and 3x standard deviations from global mean) are indicated by dotted bars.
